# Ziele und Gestaltung digitaler Plattformen für Produktionsnetzwerke

**DOI:** 10.1365/s40702-022-00908-2

**Published:** 2022-09-06

**Authors:** Chiara Freichel, Timo-Christian Steegmans, Axel Winkelmann

**Affiliations:** Lehrstuhl für BWL und Wirtschaftsinformatik, Julius-Maximilians-Universität Würzburg, Sanderring 2, 97070 Würzburg, Deutschland

**Keywords:** Digitale Plattformen, B2B, Ziele, Produktionsnetzwerke, Produktionskapazitäten, Digital platforms, B2B, Goals, Production networks, Manufacturing capacities

## Abstract

Die interorganisatorische Zusammenarbeit in Produktionsnetzwerken kann Herausforderungen durch eine hohe Marktdynamik, immer anspruchsvollere Kundenbedürfnisse und steigenden Kostendruck entgegenwirken. Neben der klassischen vertikalen Verschiebung von Kapazitäten in Richtung geeigneter Zulieferer, lassen sich Fertigungskapazitäten auch durch eine horizontale Zusammenarbeit zwischen produzierenden Unternehmen handeln. Im Sinne der Sharing Economy bieten digitale Plattformen eine geeignete Infrastruktur zur Verknüpfung und Koordination der Marktakteure eines Produktionsnetzwerks. So können Fertigungsunternehmen flexibel Produktionsausfällen entgegenwirken und freie Maschinenkapazitäten auslasten. Eine wesentliche Voraussetzung für den Erfolg solcher digitalen Plattformen für Produktionsnetzwerke ist die Definition von Zielen, welche bisher in der Literatur nur unzureichend und nicht bezogen auf diese spezifische Plattformart untersucht wurden. In dieser Arbeit wird ein umfängliches konzeptionelles Zielmodell für diese spezifische Plattformart erstellt. Zu spezifischen Zielen digitaler Plattformen für Produktionsnetzwerke zählen neben wirtschaftlichen oder technischen Zielen beispielsweise auch produktionsbezogene Marktleistungsziele wie die Gewährleistung von Produktionsflexibilität. Aufbauend darauf wird gezeigt, wie das Design der beschriebenen Plattformen einen Einfluss auf die Erreichung bestimmter Ziele hat und wie spezielle Mechanismen zur Zielerreichung beitragen.

**Zusatzmaterial online:**

Zusätzliche Informationen sind in der Online-Version dieses Artikels (10.1365/s40702-022-00908-2) enthalten.

## Abstract (en)

Interorganizational collaboration in production networks can counteract challenges posed by high market dynamics, increasingly demanding customer requirements and rising cost pressure. In addition to the vertical shift of capacities towards suitable suppliers, manufacturing capacities can also be traded through horizontal cooperation between manufacturing companies. Following the concept of the sharing economy, digital platforms offer a suitable infrastructure for connecting and coordinating the market actors in a production network. This enables manufacturing companies to flexibly counteract production downtimes and to utilize unused machine capacity. An essential requirement for the success of such digital platforms for production networks is the specification of goals, which have so far been insufficiently investigated in literature and are not specific to this type of platform. In this paper, we develop a comprehensive conceptual goal model for this specific type of platform. Specific goals of digital platforms for production networks include, in addition to economic or technical goals, production-related market service goals such as ensuring production flexibility. Based on this, we demonstrate how the design of the described platforms has an impact on the achievement of certain goals and how special mechanisms contribute to the achievement of goals.

## Motivation und Zielsetzung

Produzierende Unternehmen stehen vor der Herausforderung, eine hohe Produktionseffizienz und gleichmäßige Auslastung von Maschinen- und Anlagenkapazitäten zu gewährleisten. Verstärkt wird diese Herausforderung durch die Covid-19-Krise, welche die drastischen Einschnitte hinsichtlich der Verfügbarkeit von Produktionskapazitäten entlang der Supply Chain aufzeigt. Gleichzeitig bildet eine perfekte Kundenauftragserfüllung im Sinne der Lieferserviceziele Zeit, Zuverlässigkeit, Flexibilität und Beschaffenheit einen der wichtigsten Erfolgsfaktoren produzierender Unternehmen.

Eine Möglichkeit der Auslastung verfügbarer Kapazitäten ist die Auslagerung von vor- oder nachgelagerten Arbeiten in Richtung geeigneter Zulieferer. Man spricht an diese Stelle von der verlängerten Werkbank. Sogenannte „Single Points of Failure“ werden in einer hochspezialisierten Supply Chain jedoch nur bedingt vermieden. Neben diesem klassischen Outsourcing-Ansatz kann eine Auslastung der Produktionskapazitäten durch eine horizontale Zusammenarbeit zwischen produzierenden Unternehmen erfolgen. Bei solchen interorganisatorischen Kooperationen werden verfügbare Fertigungskapazitäten gemäß dem Konzept der „Sharing Economy“ zwischen den Akteuren im Netzwerk gehandelt. Dazu bieten digitale Plattformen eine geeignete Infrastruktur zur Verknüpfung und Koordination der Marktakteure eines Produktionsnetzwerks. Bereits auf dem Markt etabliert sind Plattformen wie *Xometry, Spanflug, Laserhub* oder *Techpilot*, welche die Anfragen und Angebote von Fertigungskapazitäten zum Handel bereitstellen und folglich ungenutzte Kapazitäten nutzbar machen.

In der bisherigen Betrachtung digitaler Plattformen für Produktionsnetzwerke bleiben die Ziele dieser spezifischen Plattformart nahezu unberücksichtigt. Einige Elemente bestehender Zielmodelle von Organisationen (Thommen et al. 2017) sowie von digitalen Plattformen im Allgemeinen (Standing et al. 2006; Williams et al. 2008; Matook 2013; Rolland et al. 2018) lassen sich auf Plattformen für Produktionsnetzwerke übertragen. Andere Aspekte, insbesondere produktionsbezogene Marktleistungsziele, wurden hingegen noch nicht untersucht oder in Zielübersichten inkludiert. Diese Entwicklung erscheint nicht nachvollziehbar, da die Bestimmung von Zielen eine wesentliche Voraussetzung für den Erfolg einer digitalen Plattform sein kann (Matook 2013). O’Reilly und Finnegan (2009) definieren die Leistung eines elektronischen Marktplatzes beispielsweise als das Ausmaß, wie effizient der elektronische Marktplatz seine Aufgaben erfüllt und seine Ziele erreicht, während er weiterhin innovativ ist, wächst und expandiert. Daraus lässt sich schließen, dass die Definition von Zielen essenziell für den Erfolg und damit für die Identifizierung verbesserungswürdiger Bereiche ist (Matook 2013; Thitimajshima et al. 2018).

Die in diesem Artikel erarbeitete übergeordnete Zielübersicht digitaler Plattformen für Produktionsnetzwerke dient der Identifikation und Systematisierung von Zielen dieser Plattformart und kann die Komplexität der Plattformziele durch die Klassifizierung in Dimensionen reduzieren (Matook 2013). Ferner fungiert die Zielübersicht als Steuerungsinstrument, welches die kontinuierliche Überwachung der Leistungsfähigkeit einer Plattform ermöglichen kann. Außerdem können Aussagen getroffen werden, welche Gestaltungselemente digitaler Plattformen für Produktionsnetzwerke dazu beitragen, definierte Ziele zu erreichen. Zur Strukturierung der vorliegenden Arbeit werden die Forschungsfragen (FF) wie folgt definiert:

### FF1

Welche Zieldimensionen und Ziele verfolgen digitale Plattformen im Allgemeinen und welche Ziele lassen sich auf digitale Plattformen für Produktionsnetzwerke übertragen?

### FF2

Welche Ziele digitaler Plattformen für Produktionsnetzwerke werden durch bestehende Zielmodelle nicht abgedeckt und wie lässt sich ein gesamtheitliches Zielmodell dieser Plattformart entwickeln?

### FF3

Wie können Empfehlungen für das Design digitaler Plattformen für Produktionsnetzwerke zur Zielerreichung abgeleitet werden?

Aufbauend auf den begrifflichen Grundlagen im nächsten Abschnitt, wird in Abschn. 3 die methodische Vorgehensweise zur Entwicklung des Zielmodells vorgestellt. Abschn. 4 stellt das erarbeitete Zielmodell digitaler Plattformen für Produktionsnetzwerke vor. Beispielhafte Empfehlungen zum Design der betrachteten Plattformen im Bezug zur Zielerreichung werden in Abschn. 5 gegeben. Ein Fazit mit Ausblick auf weitere Forschungsmöglichkeiten schließt diese Arbeit.

## Theoretische Grundlagen

### Unternehmensziele, Unternehmensnetzwerke und digitale Plattformen

Seit langem ist die Suche nach Schlüsselfaktoren zur Erreichung von *Zielen* ein bedeutsames Thema der Betriebswirtschaftslehre. Im unternehmerischen Kontext bilden Ziele einen avisierten, zukünftigen Zustand, der von den Interessensgruppen eines Betriebs bewusst angestrebt wird (Hahn 1994).

*Unternehmensziele* lassen sich in die drei Kategorien Zielinhalt, Zielausmaß und den zeitlichen Bezug gliedern (Hahn 1994). Während der Zielinhalt die Ausrichtung des unternehmerischen Handelns festlegt, ermöglicht das Ausmaß und der Zeitbezug die Operationalität der Ziele (Thommen et al. 2017). Der Fokus der vorliegenden Arbeit liegt auf den Zielinhalten, welche die konkreten Zielarten bestimmen, die sich in Sach- und Formalziele trennen (Thommen et al. 2017). Sachziele legen ihren Fokus auf konkrete Unternehmensobjekte und befassen sich mit dem Leistungserstellungsprozess. Formalziele sichern hingegen den unternehmerischen Erfolg einer Organisation (Thommen et al. 2017).

Die Definition der beschriebenen Zielkategorien steht in enger Beziehung zur *Organisationsform* eines Unternehmens und nimmt großen Einfluss auf die Erreichung der Unternehmensziele, da beispielsweise Gewinn- oder Rentabilitätsziele nicht von einem Individuum allein erreicht werden können. Netzwerke bilden ein Ökosystem, in dem Wissen, Güter und Ressourcen untereinander ausgetauscht werden können, sodass z. B. die Transparenz erhöht und eine flexible Reaktion auf neue Marktanforderungen ermöglicht wird. Ein Unternehmensnetzwerk nach Sydow (1992) stellt „eine auf die Realisierung von Wettbewerbsvorteilen zielende Organisationsform ökonomischer Aktivitäten dar, die sich durch komplex-reziproke, eher kooperative denn kompetitive und relativ stabile Beziehungen zwischen rechtlich selbständigen, wirtschaftlich jedoch zumeist abhängigen Unternehmungen auszeichnet“. Die vorliegende Arbeit stützt sich auf diese Definition und verwendet den Netzwerkbegriff unabhängig von vertraglichen Grundlagen. Konzentrieren sich die interorganisatorischen Aktivitäten in einem Unternehmensnetzwerk auf die Produktion und Produktionsprozesse, so wird von einem Produktionsnetzwerk gesprochen. Um ein Unternehmensnetzwerk erfolgreich aufzubauen, bedarf es einem geeigneten Organisationsinstrument, welches die übergreifende Kooperation zwischen Unternehmen und die damit einhergehenden Vorteile ermöglicht.

*Digitale Plattformen* sind Geschäftsmodelle zur Durchführung wertschöpfender Interaktionen zwischen Anbieter und Nachfrager (De Reuver et al. 2018). Die Plattform stellt hierbei die notwendige Infrastruktur und legt die Rahmenbedingungen für die Interaktion der Teilnehmer fest (De Reuver et al. 2018). In der Literatur finden sich unterschiedliche Sichtweisen zur Definition digitaler Plattformen. Diese Arbeit stützt sich auf die integrative Sichtweise von Gawer (2014): Demnach sind digitale Plattformen Organisationen, die:

- konstituierende Akteure, die innovativ und wettbewerbsfähig sind, zusammenschließen und koordinieren;
- Werte schaffen, indem sie Verbundvorteile bei Angebot und/oder Nachfrage generieren und nutzen und
- eine modulare technologische Architektur beinhalten, die aus einem Kern und einer Peripherie besteht.

Damit fungieren digitale Plattformen als geeignetes Instrument zur interorganisatorischen Zusammenarbeit in Produktionsnetzwerken. Neben dem Begriff der digitalen Plattform wird in der Literatur oft von elektronischen Märkten gesprochen. Gemäß den meisten identifizierten Definitionen, umfassen digitale Plattformen jedoch im Gegensatz zu elektronischen Marktplätzen häufig vielfältigere Dienstleistungen, wie beispielsweise das automatische Matching von Teilnehmern statt einer katalogbasierten Auswahl, komplette Transaktionsdienstleistungen statt reiner Vermittlung, umfangreichere Informationsaggregation, automatisierte individuelle Preisberechnung oder integrierter Datenaustausch (Zutshi und Grilo 2019). Für die vorliegende Arbeit wird daher der Begriff Marktplatz nur dann verwendet, wenn reine Handelsfunktionen beschrieben werden.

### Struktur digitaler Plattformen für Produktionsnetzwerke

Zur Beschreibung der *Struktur und des Geschäftsmodells digitaler Plattformen* werden in der Literatur eine Vielzahl von Elementen analysiert. Diese wurden basierend auf den Literaturanalysen in Freichel et al. (2021a) und Freichel et al. (2021b) sowie der in Abschn. 3 vorgestellten zusätzlichen Literaturanalyse identifiziert. Die jeweiligen Quellen finden sich im Onlinematerial 1. Für weitere detaillierte Erläuterungen kann an dieser Stelle beispielsweise auf die Modelle von Abendroth et al. (2021), Holland und Gutiérrez-Leefmans (2018), Perscheid et al. (2020), Täuscher und Laudien (2018) und Wortmann et al. (2019) sowie dort angegebene Literatur verwiesen werden. Im Folgenden werden einige in diesen Quellen genannte Gestaltungsmerkmale digitaler Plattformen für Produktionsnetzwerke zusammengestellt, die in Abschn. 5 herangezogen werden.

Zunächst unterscheiden sich die beschriebenen Plattformen für Produktionsnetzwerke hinsichtlich der *Dienstleistungsart* (Dai und Kauffman 2002; Blaschke et al. 2019). Transaktionsdienstleister wickeln die Interaktionen bzw. Transaktionen zwischen Käufern und Verkäufern komplett über eine Plattform ab, während Vermittlungsdienstleister lediglich passende Käufer und Verkäufer vernetzen (Smits und Weigand 2010). Ergänzend können zusätzliche Softwaremodule als Mehrwertdienste angeboten werden. Als weiteres Gestaltungsmerkmal lassen sich Drittanbieter, Verkäufer, Käufer oder Konsortien als *Eigentümer der Plattform* unterscheiden (Standing et al. 2006). Werden durch die Plattformbetreiber weder Käufer noch Verkäufer durch Regeln, Zugang oder Prozesse auf der Plattform bevorzugt, ist die *Marktorientierung* neutral. Verzerrte Plattformen hingegen besitzen Ausweichmechanismen, die entweder Käufer oder Verkäufer bevorzugen (Kalvenes und Basu 2006; Movahedi et al. 2012). Digitale Plattformen können sich weiterhin bezogen auf deren *Offenheit* des Marktzugangs zur Plattforminfrastruktur für verschiedene Teilnehmerarten unterscheiden (Movahedi et al. 2012; Ondrus et al. 2015; Blaschke et al. 2019). Ferner haben Plattformbetreiber die Möglichkeit, eine Eingrenzung der Akteure in Bezug auf deren *geographische Reichweite *oder angebotene/nachgefragte* Fertigungsverfahren* vorzunehmen (Täuscher und Laudien 2018). Die *Beziehungsebene* zwischen den Akteuren lässt sich durch langfristige und kurzfristige Geschäftsbeziehungen charakterisieren (Movahedi et al. 2012).

Neben den beschriebenen Unterscheidungsmerkmalen bezogen auf die Akteure lassen sich unterschiedliche Marktmechanismen abgrenzen. Dazu zählt beispielsweise die *Onlinekatalogisierung*, wobei angebotene und nachgefragte Produkte und Dienstleistungen aggregiert, kundenspezifisch oder neutral in Katalogform gelistet werden können (Dai und Kauffman 2002). Im Gegensatz zur vorgegebenen Spezifizierung durch die Plattform, können Akteure im Rahmen einer individualisierbaren* Priorisierung* eigenständig die zur Verfügung stehenden Geschäftspartner nach bestimmten Kriterien als Favoriten markieren oder ausschließen. Eine weitere wesentliche Funktion digitaler Plattformen sind *Preisermittlungsmechanismen*, welche im Unterschied zu traditionellen Märkten gänzlich neue Arten der Preisbildung ermöglichen. In der Literatur genannt werden beispielsweise die dynamische und fixe Preisermittlung (Dai und Kauffman 2002; Lu und Antony 2003; Movahedi et al. 2012, Täuscher und Laudien 2018). Alternativ ist eine manuelle Preisermittlung durch die Akteure möglich. Weiterhin lässt sich der Automationsgrad beim *Matching* passender Fertigungsverfahren und gewünschter Produkte unterscheiden.

Neben den Marktmechanismen gelten Qualitätsmanagement und Governance als wesentliche Aktivitäten der Plattformanbieter, welche den Erfolg einer Plattform bestimmen. Zunächst kann der Einsatz von Aktivitäten zur *Qualitätssicherung* der gefertigten Produkte bezogen auf den Zeitpunkt der Maßnahme (vor oder nach dem Vermittlungsprozess bzw. keine Qualitätssicherung) bestimmt werden. Weiterhin kann unterschieden werden, ob Verkäufer freiwillig oder verpflichtend eine Angabe zur eigenen *Zertifizierung* machen müssen. Unterschiedliche *Feedback-Mechanismen* umfassen die Bewertung der Akteure untereinander oder die Bewertung der Teilnehmer durch den Plattformanbieter auf der Grundlage marktgesteuerter Reputationssysteme (Täuscher und Laudien 2018). Ferner ermöglicht, wenn vorhanden, die* Führung durch Plattformbetreiber *die Ausübung zentraler Kontrollmechanismen zur Sicherung von Qualität und anderen Standards (Kazan et al. 2018). Zuletzt lassen sich digitale Plattformen hinsichtlich der *Verifizierungsmechanismen* bei der Aufnahme von Akteuren in das Netzwerk charakterisieren (Kalvenes und Basu 2006). Eine Überprüfung von Käufern umfasst beispielsweise die Zahlungsfähigkeit. Erfolgt keine Verifizierung, werden häufig auch Privatpersonen als Käufer zugelassen, wodurch der Marktplatz als offen charakterisiert werden kann.

Die nächste identifizierte Kategorie von Gestaltungsmerkmalen bildet die Plattformtechnologie, welche es ermöglicht, Transaktionen wie Käufe, Verkäufe, Vertragsabschlüsse oder die Kommunikation abzuwickeln. Während der Prozesse auf digitalen Plattformen werden eine Vielzahl von Daten erfasst, gespeichert, analysiert und interpretiert. Die *Datenhaltung* kann dabei dezentral, zentral oder einer Kombination aus beidem, d. h. hybrid, erfolgen (Perscheid et al. 2020). Die *Benutzeroberfläche* der Plattform kann entweder in Form einer reinen Webapplikation implementiert sein, anderseits in Kombination mit einer nativen (mobilen) Applikation (Täuscher und Laudien 2018). Plattformanbieter müssen weiterhin entscheiden, ob und inwiefern eine *Integration externer Systeme* der Akteure in die Plattform erfolgen kann, beispielsweise durch technische Schnittstellen und/oder elektronische Standards. Damit können Akteure z. B. Objektdateien auf der Plattform einfacher verwalten soeie freie oder belegte Produktionskapazitäten direkt an die Plattform übermitteln. Den Plattformprozessen direkt nachgelagert ist der physische Transport der produzierten Produkte durch *Logistikdienstleister*, welche zur Nutzung von Mechanismen für Preise, Lieferdauer oder Versicherung der logistischen Abwicklung in die Plattform integriert werden können (Thitimajshima et al. 2018). Weiterhin kann der Transaktionsprozess der Plattform durch die *Integration von Zahlungsdienstleistern* vereinfacht werden (Dai und Kauffman 2002).

Da es sich bei digitalen Plattformen um gewinnorientierte Unternehmen handelt, sind Plattformbetreiber letztlich auf Einnahmequellen angewiesen, um ein rentables Geschäftsmodell zu etablieren (Perscheid et al. 2020). Für Plattformbetreiber stellt sich daher die Frage, ob und wie sie von den teilnehmenden Nutzergruppen differenzierte Zugangs- und Nutzungsgebühren verlangen sollten. Unterscheiden lassen sich z. B. *wiederkehrende fixe Gebühren, transaktionsbasierte Gebühren, Inseratgebühren* oder *einmalige Gebühren *(Täuscher und Laudien 2018; Freitag et al. 2020). Mit der beschriebenen Struktur digitaler Plattformen für Produktionsnetzwerke stellt sich die Frage, woran sich die Entscheidungen für oder gegen bestimmte Elemente zur Gestaltung digitaler Plattformen zu orientieren haben, wozu im Folgenden ein Zielmodell entwickelt wird.

## Methodisches Vorgehen zur Entwicklung des Zielmodells

### Literaturanalyse

Für die Entwicklung eines untersuchungsspezifischen Zielmodells wird sich an einer Auswahl etablierter Arbeiten zu Zielarten und -anforderungen sowie organisatorischen Zielen orientiert. Jedoch werden auf Plattformen Werte auch außerhalb der Unternehmensgrenzen generiert. Der Erfolg ist also weniger von reinem Gewinn abhängig, sondern vielmehr von beispielsweise Klickraten oder erfolgreichen Transaktionen.

Zur Identifikation relevanter Ziele und Gestaltungselemente wurde eine umfassende Literaturanalyse zu digitalen Plattformen für Produktionsnetzwerke nach der schrittweisen Vorgehensweise gemäß vom Brocke et al. (2009) durchgeführt. Die Auswahl von hochqualitativen Journals und Konferenz-Proceedings beruht auf dem „VHB Jourqual3 Ranking“ mit einem Rating von A+, A und B sowie dem „Basket of Eight“ der Association for Information Systems. Die drei Datenbanken Scopus, AIS Electronic Library und EBSCO Business Source Premier wurden mit folgendem Suchterm bestehend aus den vier Begriffsgruppen „Plattform“, „Interorganisation“, „B2B und Produktion“ sowie „digital“ durchsucht:

((Ecosystem* OR Market* OR Network* OR Organi* OR Platform* OR Service*)

**OR**

(Capacit* OR Collaborat* OR Connect* OR Cooperat* OR Distribut* OR Exchang* OR Integrat* OR Interorgani* OR Match* OR* Multisided *OR* „Peer-to-Peer“ *OR* P2P *OR Shar* OR Trad*))

**AND**

*(B2B *OR *„Business-to-Business“ *OR* Business *OR Commerc* OR Econom* OR Industr* OR Logistic* OR Machine* OR Manufactur* OR* Production)*

**AND**

(Central* OR *Cloud *OR* Digital *OR* Electronic *OR* „On-Demand“ *OR* Online *OR* Smart *OR* Virtual)*

Die Suche ergab insgesamt 14.913 Suchtreffer. Nach der Titel‑, Abstract- und Volltextanalyse sowie Vorwärts- und Rückwärtssuche mit Unterstützung der Forschungsgruppe konnten für den Themenbereich digitale Plattformen für Produktionsnetzwerke 248 relevante Artikel identifiziert werden. In 53 Artikeln konnten spezifische Ziele, die für den Erfolg digitaler Plattformen entscheidend sind, identifiziert werden. Davon umfassen sechs Artikel umfängliche Zielmodelle bzw. mehrere Dimensionen. Die weiteren Artikel dienten der Erweiterung des Zielmodells um Plattformziele bezogen auf Produktionsnetzwerke sowie der Identifikation von Gestaltungselementen (bereits in Abschn. 2 vorgestellt).

### Identifikation von Kategorien und Definition der Modellstruktur

**Abb. 1:**
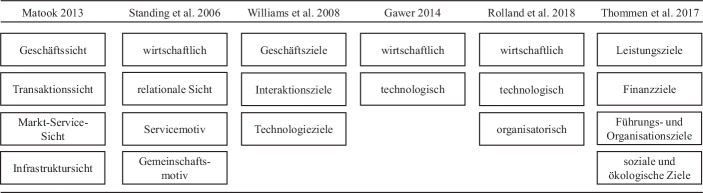
Übersicht identifizierter Zieldimensionen. (Eigene Darstellung)

Matook (2013) macht in ihrer Ausarbeitung deutlich, dass Plattformbetreiber Ziele für verschiedene strategische Bereiche festlegen und priorisieren sollten. Basierend auf der Literaturanalyse wurden die in Abb. 1 dargestellten Arbeiten mit Zieldimensionen digitaler Plattformen identifiziert. Ergänzend sind die Zieldimensionen von Thommen et al. (2017) stellvertretend für Organisationsziele im Allgemeinen aufgeführt. Aufgrund des begrenzten Umfangs dieser Arbeit kann an dieser Stelle nicht auf alle genannten Dimensionen im Detail eingegangen werden. Es wird zur detaillierten Beschreibung auf die jeweilige Literatur verwiesen. Vielmehr werden die unterschiedlichen Ansätze verglichen und argumentiert, wieso welche Dimensionen für die vorliegende Arbeit gewählt werden. Ziel ist die Beantwortung von Forschungsfrage 1.

Die Zieldimensionen nach Matook (2013) lassen sich auf die Ausführungen von Schmid und Lindemann (1998) sowie von Matook und Vessey (2008) zurückführen. Ferner stützt sich die Arbeit von Thitimajshima et al. (2018) zur Ermittlung von Faktoren, die die Leistung von elektronischen B2B-Marktplätzen beeinflussen, auf die Arbeit von Matook (2013) und wird daher nicht gesondert in Abb. 1 aufgeführt.

Nach Matook und Vessey (2008) stellt die Geschäftssicht, in der vorliegenden Arbeit bezeichnet als *wirtschaftliche Ziele*, den wesentlichen Kern eines elektronischen Marktplatzes dar. Die Geschäftssicht verdeutlicht den Zweck einer Organisation und beschreibt die Rollen, Verantwortlichkeiten und Regeln eines elektronischen Marktplatzes. Inhalte dieser Dimension sind finanzielle Aspekte, die ein nachhaltiges Wirtschaften ermöglichen sollen. Neben Matook (2013) stellen auch Williams et al. (2008) die besondere Bedeutung der Geschäftsziele heraus. Standing et al. (2006) betonen die Bedeutung wirtschaftlicher Aspekte wie Kostenreduktion, Produktivitätsvorteile oder Produkt-Markt-Strategien. Gawer (2014) präsentiert wirtschaftliche Zieldimensionen, die zahlreiche Überschneidungen zur Geschäftssicht nach Matook (2013) aufweisen. Gleiches gilt für die Finanzziele nach Thommen et al. (2017).

Eine weitere Sichtweise wird von Schmid und Lindemann (1998) als Servicesicht beschrieben. Matook und Vessey (2008) sowie Matook (2013) erweitern diesen Ansatz in der sogenannten Markt-Service-Sicht, in der vorliegenden Arbeit bezeichnet als *Marktleistungsziele*. Die Zielinhalte dieser Dimension lassen eine Verbindung zu den Leistungszielen nach Thommen et al. (2017) sowie den Interaktionszielen nach Williams et al. (2008) zu. Auch die Serviceziele nach Standing et al. (2006) können in diese Zieldimension eingeordnet werden. Der Schwerpunkt der Marktleistungsziele liegt auf der Bereitstellung eines umfassenden Serviceangebots, um neue Marktteilnehmer zu gewinnen und bestehende Nutzer zu behalten.

Matook und Vessey (2008) nennen ferner die Infrastruktursicht, in der vorliegenden Arbeit umfassender bezeichnet als *technische Ziele*. Die Infrastruktursicht stellt die Telekommunikationsbasis bereit und ermöglicht die Abwicklung von Transaktionen sowie die Einbettung weiterer Dienste. Matook (2013) stellt fest, dass die Infrastrukturziele eine technische Grundlage schaffen, die alle anderen Zielsetzungen der elektronischen Marktplätze ermöglichen. Andere Autoren, wie Williams et al. (2008) und Gawer (2014) sprechen an dieser Stelle von Technologiezielen. Inhaltlich unterscheiden sich die Aussagen der Autoren unwesentlich, weshalb eine Zusammenführung der Ansätze in einer technischen Dimension sinnvoll erscheint. So wird in den technischen Zielen auch die Transaktionssicht nach Matook und Vessey (2008) bzw. Matook (2013) mit Fokus auf die Verbindung zwischen dem Geschäftsmodell und den Akteuren des elektronischen Marktes inkludiert. Ferner lassen sich die Interaktionsziele nach Williams et al. (2008), die sich auf die Mensch-Computer-Interaktionen beziehen, zu den technischen Zielen hinzufügen. Inhaltlich folgen alle Ziele der breiten Definition nach Gawer (2014), wonach Plattformen eine technische Grundlage schaffen, die Innovationen ermöglichen.

*Organisatorische Ziele* werden in der Zielübersicht von Matook (2013) nicht berücksichtigt. Ausschließlich Thommen et al. (2017) und Rolland et al. (2018) stellen organisatorische Aspekte in einer eigenen Dimension dar. Thommen et al. (2017) beschreiben in dieser Zielkategorie die Ziele eines Unternehmens hinsichtlich der Unternehmensorganisation und -struktur. Den Ausführungen der Autoren folgend wird auch im Rahmen dieser Zielübersicht eine organisatorische Zieldimension etabliert.

Die letzte Zieldimension wird durch *soziale und ökologische Ziele* repräsentiert. Ähnliche Dimensionen präsentieren Thommen et al. (2017) in ihren sozialen und ökologischen Zielen. Auch die relationalen und Gemeinschafts-Ziele nach Standing et al. (2006) lassen sich dieser Dimension inhaltlich zuweisen. Die Inhalte legen das Augenmerk auf die Arbeitnehmer und die gesellschaftliche Verantwortung einer Organisation.

Auch wenn beispielsweise die Übersicht nach Matook (2013) viele Aspekte einer Zielübersicht digitaler Plattformen berücksichtigt, ist keine organisatorische und sozio-ökologische Dimension vorhanden. Die Zielübersicht der vorliegenden Arbeit wird entsprechend angepasst und umfasst in diesem Schritt *wirtschaftliche Ziele, Marktleistungsziele, technische Ziele, organisatorische Ziele* sowie *soziale & ökologische Ziele*.

### Identifikation und Kategorisierung konkreter Zielinhalte

Aufbauend auf den vier Kategorien nach Matook (2013) wurden im nächsten Schritt konkrete Zielinhalte aus der identifizierten Literatur analysiert und kategorisiert. Im Rahmen eines iterativen Verfahrens wurden durch drei Experten inhaltlich zusammengehörige Einzelziele zu ihren jeweiligen Kategorien „bottom-up“ gebündelt. Zudem wurden die Dimensionen anhand fachlich-logischer Überlegungen mittels eines „top-down“-Ansatzes in Unterziele aufgespalten. Dies ist ein teilweise unvermeidbar subjektives Vorgehen. Die Ergebnisqualität lässt sich aber durch Berücksichtigung entscheidungstheoretischer Anforderungen an Zielsysteme erhöhen (Eisenführ et al. 2010). Entsprechend wurde ein besonderes Augenmerk auf die dort genannten Forderungen gelegt. Dazu zählen beispielsweise die vollständige Abbildung und Redundanzfreiheit des Zielraums. Dazu zeigt die Konzeptmatrix in Tab. 1 das Ergebnis der Auswertung. Die zugehörigen Quellen finden sich im Onlinematerial 2.

**Tab. 1:** Konzeptmatrix zur Entwicklung des Zielmodells. (Eigene Darstellung)

	Wirtschaftliche Ziele					Marktleistungsziele				Technische Ziele				Organisatorische Ziele				Soziale & ökologische Ziele			
Artikel	Gewinn	Wachstum	Nutzeranzahl	Transaktionsanzahl, -vol	Marktanteil	Nutzerservice	Nutzertreue	Sicherheit & Datenschutz	Transaktionsservice	Nutzerfreundlichkeit	Zuverlässigkeit	Hard- & Softwarequalität	Interoperabilität	Governance	Zugänglichkeit	Agilität	Neutralität	Vertrauen	Nachhaltigkeit	Reputation	Nutzerzufriedenheit
Alt und Klein (2011)			●	●				●						●							
Asadullah et al. (2018)		●											●	●					●		
Bai und Velamuri (2021)									●					●							
Bakos (1991)				●		●	●		●												
Bakos (1998)	●								●					●							
Blaschke und Brosius (2018)													●		●	●					
Böhme und Koble (2007)								●													
Broekhuizen et al. (2021)															●						
Bunduchi (2008)									●									●			
Chen et al. (2016)											●										
Croitor und Adam (2020)														●							
Dai und Kauffman (2002)										●			●								
Datta und Chatterjee (2008)																		●			
De Reuver et al. (2018)													●	●	●	●					
Dou und Chou (2002)			●																		
Easton und Araujo (2003)				●		●					●	●									
Grewal et al. (2010)					●									●							
Gunasekaran et al. (2002)				●	●			●													
Hawlitschek et al. (2016)																		●			
Howard et al. (2006)														●	●		●				
Janita und Miranda (2013)							●	●													●
Kajan und Stoimenov (2005)													●								
Kalvenes und Basu (2006)								●													
Kim und Ahn (2007)																		●		●	
Mahadevan (2003)																	●				
Mahalingam et al. (2020)														●							
Mallapragada et al. (2015)																					●
Matook (2013)	●	●		●		●	●		●	●		●			●	●			●	●	●
Möhlmann (2021)																		●			
Ondrus et al. (2015)			●		●					●			●								
Oppong-Tawiah et al. (2020)		●											●								
Ordanini et al. (2004)			●	●									●				●				
Pavlou und Gefen (2004)							●											●			
Petersen et al. (2007)			●			●													●		
Pressey und Ashton (2009)			●														●				
Rolland et al. (2018)												●									
Saadatmand et al. (2017)													●								
Smits und Weigand (2010)				●					●			●			●		●				
Standing und Lin (2007)						●						●									
Standing et al. (2006)											●	●			●	●		●			●
Standing et al. (2010)														●						●	
Suh und Houston (2010)																		●		●	
Sun (2010)																		●			
Thitimajshima et al. (2018)			●		●	●	●	●	●	●		●						●		●	
Tuma (1998)																●					
Veisdal (2020)			●																		
Wang et al. (2008)														●							
Wang et al. (2011)						●										●					●
Wang et al. (2018)							●	●												●	
Wang et al. (2019)											●									●	●
Williams et al. (2008)	●	●				●				●		●				●					
Yoo et al. (2007)													●				●				
Zutshi und Grilo (2019)						●									●						

Alle Quellen zu Tab. 1 finden sich im Onlinematerial 2

Erneut liefert die Arbeit von Matook (2013) einen großen Beitrag zum Zielmodell des vorliegenden Artikels. Matook (2013) stützt sich in einem mehrstufigen Prozess zur Identifikation der Ziele auf bestehende Literatur sowie auf Aussagen von Experten aus dem Fachbereich elektronischer Marktplätze. Neben Matook (2013) stellen zahlreiche andere Autoren mögliche Ziele digitaler Plattformen vor. Durch die Zusammenführung der Erkenntnisse nach Matook (2013) mit weiteren Zielen digitaler Plattformen wurde die Zielübersicht für digitale Plattformen in den ersten beiden Zeilen in Tab. 1 entwickelt. Dabei entsprechen die Kategorien in Tab. 1 bereits den in Abschn. 3.2 erarbeiteten Zielkategorien.

### Anwendung auf das Anwendungsfeld digitaler Plattformen für Produktionsnetzwerke

Die Analyse der sich auf die Strukturelemente beziehenden Literatur zeigt, dass digitale Plattformen für Produktionsnetzwerke individuelle Merkmale aufweisen, die sich zum Teil von den Merkmalen allgemeiner digitaler Plattformen unterscheiden. Ein Zielmodell, welches sich vollumfänglich auf digitale Plattformen für Produktionsnetzwerke anwenden lässt und dabei alle Ziele abdeckt, konnte in den untersuchten Arbeiten nicht identifiziert werden. Demgegenüber steht die Auffassung, dass Produktionsplattformen den digitalen Plattformen untergeordnet und demnach eine Ausprägungsform digitaler Plattformen darstellen. In diesem Zusammenhang liegen Überschneidungen der beiden Plattformarten nah. Resultierend daraus wird angenommen, dass grundsätzliche Gemeinsamkeiten zwischen den Zielen digitaler Plattformen und den Zielen digitaler Produktionsplattformen vorliegen und die identifizierten Dimensionen und Zielinhalte als Grundlage für das Zielmodell dienen.

Die bestehende Zielübersicht digitaler Plattformen wurde darauf aufbauend mit Erkenntnissen aus Artikeln des Themenfeldes Produktion, Produktionsnetzwerke und Produktionsplattformen angereichert. Die Autoren dieser Artikel unterstützen zum Teil eine direkte Übertragung auf Produktionsplattformen. Andere Aussagen weisen auf notwendige Anpassungen in der Zielübersicht hin. Die aus der Literatur abgeleitete Zielübersicht wurde somit durch die Dimension *produktionsbezogene Marktleistungsziele *ergänzt, die bestehende Dimension Marktleistungsziele wurde umbenannt zu *plattformbezogenen Marktleistungszielen*. Der angebotene Service einer Produktionsplattform lässt sich folglich granularer definieren als das allgemeine Serviceangebot digitaler Plattformen. Plattformbezogene Marktleistungsziele verweisen auf Ziele unmittelbar bezogen auf Transaktionen und Nutzeraktivitäten auf der Plattform. Produktionsbezogene Marktleistungsziele umfassen hingegen Ziele, die das gehandelte Produkt der Plattform thematisieren, d. h. insbesondere die Produktion. Sie spiegeln also gleichzeitig die primären Anreize produzierender Unternehmen zur Nutzung der Plattformen wider.

## Vorstellung des Zielmodells

**Abb. 2:**
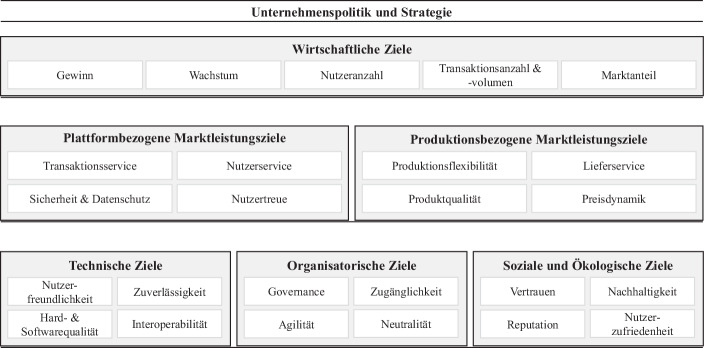
Zielmodell digitaler Plattformen für Produktionsnetzwerke. (Eigene Darstellung)

Das entwickelte Zielmodell dient der Definition und Klassifizierung von Zielen digitaler Plattformen für Produktionsnetzwerke und beantwortet damit Forschungsfrage 2. Das finale Zielmodell ist in Abb. 2 dargestellt. Dabei beinhalten die wirtschaftlichen Ziele sowohl die Ziele der Leistungserstellung und -verwertung als auch alle kosten- bzw. finanzwirtschaftlich-bezogenen Ziele. Die weiteren Zielkategorien beschreiben lediglich die Leistungsseite der Ziele, da die Kostenseite jeweils das Ziel geringer Kosten für die Leistungserfüllung verfolgt (z. B. vollständige Gewährleistung des Datenschutzes *mit möglichst geringem Kostenaufwand*). Die Ziele sind nicht überschneidungsfrei. In dieser Arbeit wird aufgrund des begrenzten Umfangs nicht auf die Beeinflussung der Ziele untereinander (z. B. Verzicht auf Gewinn, dafür Erreichung einer kritischen Masse) eingegangen.

### Wirtschaftliche Ziele

Wirtschaftliche Ziele sind den anderen Zielkategorien übergeordnet und daher entsprechend im Framework angeordnet (Matook und Vessey 2008; Williams et al. 2008).

Das Erzielen von *Gewinn*, d. h. Ertragsüberschuss, ist für Unternehmen eine wesentliche Voraussetzung, um langfristig Liquidität zu gewährleisten und erfolgreich zu sein (Thommen et al. 2017). Gewinn bezeichnet die Differenz zwischen Ertrag und Kosten. Zur Analyse der Zielerreichung sind Ertrag und Kosten (z. B. für Transaktionen oder Betrieb) gleichermaßen zu betrachten, da Unternehmen häufig bewusst ihre Kosten steigern, um Differenzierung durch höhere Preise zu erzielen (Porter 2014).

Das *Wachstum* digitaler Plattformen für Produktionsnetzwerke kann klassisch über die Zunahme von Umsatz beschrieben werden. Das Wachstum im Sinne von Umsatzzunahme wird jedoch häufig nicht isoliert betrachtet. Allgemein lassen sich die erfolgreiche Etablierung einer Marke, Steigerung der organisationalen Wissensbasis sowie Erhöhung der systemischen Intelligenz mit einbeziehen (Williams et al. 2008). Durch ein verbessertes Leistungsangebot steigt der Nutzen für die Plattformteilnehmer. Dies wiederum resultiert in steigender Nachfrage durch neue Teilnehmer, wodurch Plattformbetreiber Umsätze steigern können. In der vorliegenden Arbeit repräsentiert die Umsatzsteigerung das Ziel Wachstum.

Plattformen können einerseits eine hohe *Nutzeranzahl* anstreben. Andererseits kann ein kleines, geschlossenes Netzwerk als Ziel gesetzt werden. In beiden Fällen ist jedoch das Erreichen und Halten einer kritischen Masse aktiver Nutzer für den Betrieb der Plattform erforderlich (Matook 2013). Eine hohe Nutzeranzahl schafft wiederum positive Netzwerkeffekte (Thitimajshima et al. 2018).

Weiterhin beeinflussen die Höhe von *Transaktionsanzahl und -volumen* je Teilnehmer den wirtschaftlichen Erfolg der Plattformen (Matook 2013; Thitimajshima et al. 2018). Unter einer Transaktion wird der komplette Prozess des Kaufs bzw. Verkaufs von Fertigungskapazitäten oder die erfolgreiche Vermittlung zwischen Käufer und Verkäufer bezeichnet. Das Transaktionsvolumen definiert Matook (2013) als den Gesamtwert der Transaktionen innerhalb eines Zeitraums.

Der *Marktanteil* als weiteres Ziel beschreibt die Wettbewerbsposition des Plattformunternehmens. Dabei lassen sich der absolute und relative Marktanteil abgrenzen. Der absolute Marktanteil beschreibt den monetären oder volumenbezogenen Anteil (Umsatzanteil) der Plattform am gesamten Marktvolumen aller Plattformen für Produktionsnetzwerke. Der relative Marktanteil hingegen vergleicht den Marktanteil der betrachteten Plattform mit dem seines größten Konkurrenten bzw. am kombinierten Marktanteil mehrerer führender Wettbewerber. Der Marktanteil wird häufig mit der Marktmacht und finanziellen Leistung in Verbindung gebracht und ist daher ein bedeutsames wirtschaftliches Ziel.

### Plattformbezogene Marktleistungsziele

Marktleistungsziele beziehen sich auf die Bereitstellung eines umfassenden Leistungs- und Serviceangebots. Zunächst werden in diesem Abschnitt die plattformbezogenen Marktleistungsziele betrachtet.

Digitale Plattformen zum Handel von Produktionskapazitäten schaffen Anreize zur Nutzung u. a. durch schnelle Suche und Vergleich, passende Vermittlung und Verhandlung sowie einfache Koordinierung von Anbietern und Nachfragern. Sie steigern folglich Umfang und Qualität des *Transaktionsservice* für die Plattformteilnehmer, d. h. die Performance des kompletten Kauf‑, Verkauf- oder Vermittlungsprozesses, ggfs. inklusive Qualitätssicherung, Zahlungsabwicklung oder Lieferung (Täuscher und Laudien 2018).

Das weitere Ziel *Sicherheit & Datenschutz* gilt als wesentlicher Bestandteil des elektronischen Service auf Plattformen (Janita und Miranda 2013). Das Ziel beinhaltet den Schutz von persönlichen Informationen, Unternehmensdaten und transaktionsbezogenen Informationen der Nutzer.

Die Qualität und der Umfang des *Nutzerservice* hat gemäß Matook (2013) einen wesentlichen Einfluss auf das Fortbestehen oder Scheitern digitaler Plattformen. Der Nutzerservice beschreibt die Erfüllung von Erwartungen der Plattformnutzer durch einen Kommunikationsprozess zwischen der Plattform und den Teilnehmern. Zum Nutzerservice zählen sekundäre Leistungsbündel ergänzend zum primären Transaktionsprozess, wie insbesondere die Aggregation von Informationen zu Verkäufern, Preisen und Produktionsspezifika (Zutshi und Grilo 2019).

Die *Nutzertreue* repräsentiert ein weiteres plattformbezogenes Marktleistungsziel. Die Bindung von Nutzern führt zu wiederholtem Kauf- und Verkaufsverhalten. Im Fokus dieser Arbeit steht die faktische, vom Plattformbetreiber ausgehende Kundenbindung. Dazu zählt insbesondere die Umsetzung von Lock-in Effekten, in welchen Kunden durch Wechselbarrieren der Plattform treu bleiben. Die vom Kunden ausgehende, psychologische Nutzertreue im Sinne von Engagement als Grad der freiwilligen Zuweisung von persönlichen, kognitiven, emotionalen und verhaltensbezogenen Ressourcen (Yu und Ramaprasad 2019) wird hingegen weitgehend von anderen Zielen geprägt.

### Produktionsbezogene Marktleistungsziele

Die weitere Kategorie der Marktleistungsziele umfasst Ziele zum Leistungs- und Serviceangebot bezogen auf die Produktion bzw. Produkte, welche über die Plattform gehandelt werden.

Ein wesentlicher Anreiz zur Nutzung digitaler Plattformen zum Handel von Produktionskapazitäten ist die *Produktionsflexibilität*. Flexibilität ist charakterisiert durch die Anpassungsfähigkeit an Umweltveränderungen durch vorhandene Entscheidungsspielräume. Durch den einfachen Zugang zu Käufern und Verkäufern, können Produktionsunternehmen schnell und einfach auf Angebots- und Nachfrageschwankungen reagieren. Dabei werden individuelle Kapazitätserweiterungen der Produktionsunternehmen durch die entstehende Flexibilität im Produktionsnetzwerk vermieden (Freitag et al. 2020).

Auch die *Produktqualität* kann durch digitale Plattformen für Produktionsnetzwerke verbessert werden. Die meisten Plattformen vermitteln lediglich Käufer und Verkäufer von Fertigungskapazitäten oder agieren als Zwischenhändler, d. h. sie fertigen als Plattform-Unternehmen nicht selbst. Durch das Leistungsspektrum der Plattform, wie beispielsweise die breite Auswahl zwischen Lieferanten, Überprüfung der Produktionsdaten oder einheitliche Qualitätsstandards, kann eine höhere Produktqualität erreicht werden.

Weiterhin streben digitale Plattformen eine hohe Qualität des *Lieferservice* zum Kunden an. Die Qualität des Lieferservice setzt sich generell zusammen aus der Qualität von Lieferzeit, -zuverlässigkeit, -beschaffenheit und -flexibilität. Es ist davon auszugehen, dass die Auslieferung der Produkte selten Kernkompetenz der Plattformbetreiber ist. Dennoch liegt der Lieferservice als Teil des gesamten Handelsprozesses im Verantwortungsbereich der Plattform.

Durch *Preisdynamik *ermöglichen digitale Plattformen, das Angebot und die Nachfrage nach Produktionskapazitäten marktgerecht zu steuern. Die Dynamik der Preise beschreibt anhand einer Reihe von vordefinierten Regeln, wie fein abgestimmt ein Preis auf Produkt und Zielgruppe ist. Dabei ist der Preis für die jeweiligen Fertigungskapazitäten getrieben von Faktoren wie zeitliche Flexibilität von Käufer und Verkäufer, aktuelle Materialpreise oder verfügbare Kapazitäten.

### Technische Ziele

Angelehnt ist die Kategorie der technischen Ziele an die Infrastrukturziele nach Matook (2013), die Technologieziele gemäß Williams et al. (2008) und die technische Entwicklungsperspektive nach Gawer (2014).

Die *Nutzerfreundlichkeit* bezeichnet die einfache, auf den Nutzer und seine Aufgaben abgestimmte Interaktion mit der Plattform. Ferner lässt sich die Nutzerfreundlichkeit von Websites digitaler Plattformen als das Ausmaß definieren, in dem eine Website von Kunden genutzt werden kann, um bestimmte Ziele effektiv, effizient und zufriedenstellend zu erreichen (Lee und Kozar 2012). Zur Zielerreichung zählen Aspekte wie Einfachheit, Lesbarkeit, Konsistenz, Interaktivität, Navigation oder Relevanz der Inhalte (Lee und Kozar 2012).

Wesentlich für den Betrieb digitaler Plattformen ist eine hohe *Hard- und Softwarequalität*. Matook (2013) definiert die Hard- und Softwarequalität als das Ausmaß, in dem ein Teilnehmer den Eindruck hat, dass die Hard- und Software sowie andere technische Ausrüstung die gewünschten Eigenschaften besitzen und die Erwartungen der Nutzer erfüllen. Wesentliche Einflussfaktoren auf die Hard- und Softwarequalität sind deren Wartbarkeit, Skalier- und Entwicklungsfähigkeit (Rolland et al. 2018).

Als weiteres Ziel wurde die *Zuverlässigkeit* identifiziert. Dieses Ziel beschreibt die fehlerfreie technische Funktion digitaler Plattformen (Thitimajshima et al. 2018). Zuverlässigkeit bezeichnet die Fähigkeit, die versprochene Leistung genau und rechtzeitig zu erbringen. Die Zugänglichkeit der Website sowie Kompatibilität zwischen Browser und Plattform beeinflussen die technische Zuverlässigkeit.

Abschließend wurde die *Interoperabilität* als bedeutsames Ziel dieser Kategorie identifiziert (Freitag et al. 2020). Interoperabilität ist definiert als die technologische Fähigkeit unterschiedlicher Systeme oder Techniken, möglichst nahtlos zusammenzuarbeiten (Ondrus et al. 2015). Interoperabilität zeichnet sich insbesondere durch die Implementierung einheitlicher Schnittstellen und elektronischer Standards aus (De Reuver et al. 2018). Durch einheitliche Datenmodelle und -formate einerseits und die Angleichung von Geschäftsprozessen andererseits, lassen sich Systeme und Prozesse der Plattformteilnehmer integrieren (Dai und Kauffman 2002).

### Organisatorische Ziele

Thommen et al. (2017) sowie Rolland et al. (2018) betrachten organisatorische Aspekte als eigene Kategorie. Die Organisation wird generell von Prozessen und Strukturen bestimmt. Diese sollten so gewählt werden, dass die unternehmerischen Aufgaben bestmöglich erfüllt werden können.

*Governance* bezeichnet allgemein das Steuerungs- und Regelungssystem von Organisationen. Das Ziel Plattform-Governance bezieht sich auf die Orchestrierung der Interaktion verschiedener Akteure. Die Umsetzung der drei Governance Aspekte Entscheidungsrechte, Kontrollmechanismen und Anreizstrukturen tragen wesentlich zum Erfolg der Plattform bei.

Das Ziel *Agilität* wird als höchste Form der Anpassungsfähigkeit beschrieben und ermöglicht die schnelle Schaffung von Innovationen. Agilität zeichnet sich durch proaktiv, antizipativ und initiative organisatorische Anpassung der digitalen Plattformen aus.

Ein weiteres Ziel der Dimension organisatorischer Ziele ist die *Zugänglichkeit*. Zugänglichkeit bezieht sich im Gegensatz zur Interoperabilität auf organisatorische, statt technische Ein- und Ausgangsregeln. Zusammenfassend definiert Matook (2013) Zugänglichkeit als die Verfügbarkeit und Geschwindigkeit des Zugangs zur digitalen Plattform. Der Zugang ist durch den Grad der Offenheit der Plattform charakterisiert.

Das Ziel der *Neutralität* wird durch das Wettbewerbsverhältnis und die Rolle des Plattformeigentümers bestimmt. Neutralität bedeutet, dass keine Partei der Plattformteilnehmer, d. h. Käufer oder Verkäufer bevorzugt werden. Agieren die Plattformen unabhängig und unparteiisch, kann eine gerechte Verteilung von Angebot und Nachfrage gewährleistet und Konkurrenzverhältnisse verhindert werden (Smits und Weigand 2010).

### Soziale und ökologische Ziele

Zusammengefasst wurde diese Kategorie aus den sozialen und ökologischen Zielen gemäß Thommen et al. (2017) und relationalen und Gemeinschaftszielen nach Standing et al. (2006). Die Kategorie integriert die gesellschaftliche, ökologische und ökonomische Perspektive. Während diese Zielkategorie in der Literatur oftmals das Augenmerk auf die Mitarbeiter der Unternehmen legt (Thommen et al. 2017), stehen in der vorliegenden Arbeit die Teilnehmer der digitalen Plattform im Fokus.

Das *Vertrauen* in digitale Plattformen und deren Plattformbetreiber bildet eine wesentliche Grundlage für Interaktionen auf der Plattform (Zutshi und Grilo 2019). Durch Vertrauen der Teilnehmer wird ein reibungsloser Ablauf von Transaktionen gewährleistet. Weiterhin ermöglichen Vertrauensbeziehungen zwischen den Nutzern und dem Plattformbetreiber Integrität der Plattform.

Das Ansehen bzw. der Wiedererkennungswert digitaler Plattformen bei aktuellen und potenziellen Teilnehmern wird als *Reputation* bezeichnet. Der Plattformbetreiber erhält Auskunft darüber, wie die Plattform von den Nutzern wahrgenommen wird und kann seinen Bekanntheitsgrad erhöhen. Das Management von Reputation trägt zur Erreichung des Ziels bei.

Ferner ist die *Nachhaltigkeit* ein Bestandteil dieser Kategorie. In dieser Arbeit bezieht sich das Ziel Nachhaltigkeit sowohl auf das Handeln des Plattformunternehmens als auch auf die Auswirkungen des Geschäftsmodells. Nachhaltigkeit beschreibt langfristig erfolgreiches Handeln nach ökonomischen, sozialen und ökologischen Gesichtspunkten und verbindet damit verschiedene Ziele (Matook 2013; Thommen et al. 2017). Aufgrund der zahlreichen Sichtweisen und Aspekte trägt eine Schwerpunktsetzung der Plattformbetreiber zur Zielerreichung bei. Im Vordergrund steht daher für die vorliegende Arbeit die ökologische Nachhaltigkeit, welche neben der wirtschaftlichen Nachhaltigkeit zunehmend an gesellschaftlicher Relevanz gewinnt.

Abschließend wird das Ziel *Nutzerzufriedenheit* vorgestellt. Digitale Plattformen für Produktionsnetzwerke dienen der Befriedigung personeller Bedürfnisse und Motive der Teilnehmer. Gemäß der Definition von Matook (2013) beschreibt die Zufriedenheit eine positive Reaktion eines Teilnehmers auf das gesamte Transaktionserlebnis auf der Plattform. Es ist zur Zielerreichung von großer Bedeutung, zu verstehen, wie Nutzer zufrieden gestellt werden können.

## Gestaltungsmöglichkeiten zur Zielerreichung

Dieses Kapitel dient der Anwendung und Demonstration des Zielmodells anhand der Ableitung von Empfehlungen für das Design digitaler Plattformen für Produktionsnetzwerke zur Zielerreichung. Beantwortet wird entsprechend Forschungsfrage 3. Die beschriebenen Strukturmerkmale in Abschn. 2 dienen als Grundlage und werden nun in Verbindung mit dem Zielmodell gesetzt. Die im Folgenden vorgestellten Gestaltungsmöglichkeiten zur Zielerreichung wurden entlang der Ergebnisse der Literaturanalyse sowie durch Workshops mit mehreren Experten der Forschungsgruppe konzeptionell erarbeitet. Im Rahmen dieses Prozesses bewerteten die drei Experten unabhängig voneinander, ob die jeweiligen Strukturelemente einen positiven Einfluss auf die jeweiligen Ziele des Zielmodells haben. Die Beispiele in diesem Kapitel entsprechen den wesentlichen Einflüssen, in welchen keine Abweichungen zwischen den Ergebnissen der Experten bestehen. Es wird kein Anspruch auf ein vollständiges Struktur-Ziel-Einflussmodell erhoben. Vielmehr werden beispielhafte Gestaltungsmöglichkeiten zur jeweiligen Zielerreichung erarbeitet, um die Anwendung des Zielmodells zu demonstrieren.

### Gestaltungsmöglichkeiten zur Erreichung wirtschaftlicher Ziele

Als übergeordnete Zieldimension werden wirtschaftliche Ziele von fast allen Strukturelementen beeinflusst. Lediglich die Marktmechanismen auf der Handelsplattform haben wenig Einfluss auf die wirtschaftlichen Ziele. Angebotene Mehrwertdienste wirken sich positiv auf Umsatz, Nutzeranzahl und Transaktionsvolumen aus und damit auch auf den Marktanteil.

Bei der Wahl der Akteure zeichnet sich ein einfaches Bild ab. Eine möglichst offene Plattform, die möglichst weltweit agiert und für alle Fertigungsverfahren offen ist, hat das größte Potenzial zu wachsen, adressiert viele Nutzer und kann dadurch ein großes Transaktionsvolumen ermöglichen. Eine Einschränkung des Marktes auf bestimmte Regionen oder Fertigungsverfahren definiert den Markt, auf dem die Plattform agiert und kann daher einen direkten Einfluss auf den Marktanteil haben.

Die Marktmechanismen haben wenig Einfluss auf die wirtschaftlichen Ziele. Jedoch lassen sich insbesondere Verfahren für dynamisches Pricing herausstellen, die zum Ziel haben, das Transaktionsvolumen auf Marktplattformen zu maximieren. Ein manuelles Matching erzeugt auf Dauer hohe Kosten für den Plattformbetreiber und kann durch den manuellen Aufwand das maximale Transaktionsvolumen beschränken. Qualitätsmanagement und Governance sind meist mit hohen Kosten verbunden und schmälern dadurch den Gewinn. Die nachgelagerten positiven Effekte machen sich erst im Laufe der Zeit bemerkbar. Viele der Maßnahmen hindern zudem neue Nutzer daran, am Markt teilzunehmen, oder steuern und verbieten bestimmte Transaktionen.

Auch bei der Plattformtechnologie sind die meisten Maßnahmen mit hohen Kosten verbunden, die sich auf den Gewinn auswirken. Jedoch wirken sich insbesondere Maßnahmen, die Mehrwerte für den Nutzer bieten, positiv auf Wachstum, Nutzeranzahl und Transaktionsvolumen aus.

Bei den Einnahmequellen wirken sich insgesamt viele unterschiedliche Gebühren positiv auf den Gewinn aus. Einmalige Einrichtungsgebühren oder Abonnementgebühren können allerdings neue Nutzer abschrecken und fernhalten. Transaktionsbasierte Gebühren oder Inseratgebühren können wiederum bestehende Kunden daran hindern, gerade kleinere Transaktionen durchzuführen und schmälern somit das Transaktionsvolumen und in der Folge wiederum den Marktanteil. Diese Beziehungen sind mehrstufig und bedürfen einer feinen Abstimmung des gesamten Gebührenmodells.

### Gestaltungsmöglichkeiten zur Erreichung plattformbezogener Marktleistungsziele

Die plattformbezogenen Marktleistungsziele werden stark durch den Dienstleistungsumfang bestimmt. Sowohl die Art der Dienstleistung, d. h. ob komplette Transaktionen von der Plattform abgewickelt werden oder die Plattform nur als Vermittler agiert, als auch die angebotenen Mehrwertdienste haben einen direkten Einfluss auf die Zielerreichung. Lediglich das Ziel Sicherheit & Datenschutz wird vom Dienstleistungsumfang nicht beeinflusst.

Die Gestaltung der Akteure auf der Plattform hat nur einen beschränkten Einfluss auf die Marktleistungsziele. Der Transaktionsservice und Nutzerservice wird positiv durch ein breiteres Angebot an Fertigungsverfahren beeinflusst. Langfristige Beziehungen erhöhen die Nutzertreue. Ferner wird das Ziel Sicherheit & Datenschutz nur indirekt durch Offenheit, geographische Reichweite und ein großes Angebot an Fertigungsverfahren negativ beeinflusst. Diese sorgen für eine größere Nutzerbasis und damit ein höheres Risiko für Datenschutzvorfälle. Die Plattform kann resultierend daraus als ein lukrativeres Ziel für Cyberattacken dargestellt werden.

Sämtliche Gestaltungselemente der Marktmechanismen haben einen Einfluss auf den Transaktionsservice, da diese durch zusätzliche Funktionalitäten die Transaktionen vereinfachen oder beschleunigen. Die Möglichkeit für Nutzer, entsprechende Käufer und Verkäufer zu priorisieren, stärkt zudem die Nutzertreue. Onlinekatalogisierung und automatisiertes Matching sind nur möglich, wenn die Plattform über die notwendigen Daten verfügt, was wiederum ein mögliches Angriffsziel darstellt.

Im Bereich Qualitätsmanagement beeinflussen die meisten Gestaltungselemente die plattformbezogenen Marktleistungsziele. Insgesamt wirken sich klare und umfangreiche Governance-Maßnahmen positiv auf die Nutzertreue aus. Zudem verbessert insbesondere die Führung durch den Plattformbetreiber mittels Durchsetzungs- und Schlichtungsmaßnahmen alle Serviceziele sowie die Sicherheit und Nutzertreue.

Auch die Plattformtechnologie hat einen großen Einfluss auf die Erreichung der Marktleistungsziele. Während eine zentrale Datenhaltung mehr Informationen bündelt und somit einen besseren Transaktions- und Nutzerservice und damit eine hohe Nutzertreue ermöglicht, so erhöht sich gleichzeitig auch das Risiko, dass zentral gelagerte Daten missbraucht werden. Gleichermaßen verhält sich die Integration externer Systeme. Bei der Integration von externen Dienstleistern muss beachtet werden, dass diese zwar den Transaktionsservice um weitere Dienstleistungen erweitern, dadurch jedoch als Teil des solchen bewertet werden müssen. Eine Integration kann sich, wenn sie schlecht umgesetzt wird, daher auch negativ auf den gesamten Service auswirken.

Bei den Einnahmequellen lässt sich feststellen, dass einmalige Einrichtungsgebühren und Abonnementgebühren durch einen „Lock-In-Effekt“ die Nutzertreue erhöhen können.

### Gestaltungsmöglichkeiten zur Erreichung produktionsbezogener Marktleistungsziele

Ähnlich wie bei den plattformbezogenen Marktleistungszielen werden die produktionsbezogenen Marktleistungsziele hauptsächlich durch die zielgerechte Gestaltung von Qualitätsmanagement und Governance erreicht.

Bei der Wahl der Akteure auf dem Marktplatz ist ein möglichst offener und globaler Marktplatz, der viele Fertigungsverfahren anbietet, hilfreich, um die Produktion über eine größere Auswahl an Akteuren flexibel zu steuern. Hier wirkt sich auch eine kurzfristige Beziehungsebene positiv auf die Produktionsflexibilität aus. Wenn der Eigentümer der Plattform auch gleichzeitig Teilnehmer ist, kann er flexibel einspringen, um wichtige Marktteilnehmer schnell und mit guter Qualität zu bedienen. Diese Flexibilität kann zwar kurzfristig auf Kosten der eigenen Produktion gehen, jedoch besteht hier der Anreiz, Teilnehmer langfristig im Netzwerk zu halten.

Fast alle Marktmechanismen beeinflussen die Flexibilität der Produktion. Einschränkungen wie ein kundenspezifischer Onlinekatalog oder sogenanntes „Blacklisting“ von anderen Teilnehmern wirken sich negativ auf die Flexibilität aus. Ein dynamisches Pricing kann hingegen Anreize schaffen, auch bei großer Auslastung Kapazitäten anzubieten.

Bei den Qualitäts- und Governance-Maßnahmen sind insbesondere für Produktionsflexibilität und Produktqualität gegenläufige Effekte zu beobachten. Fast alle Maßnahmen schränken die Flexibilität ein, um eine hohe Qualität und einen guten Lieferservice zu ermöglichen. Dies geschieht durch das Ausschließen von Akteuren wegen fehlender Zertifizierungen oder mangelhafter ex-ante Qualitätsprüfung. Lediglich die Feedback-Mechanismen haben keinen direkten Einfluss auf die Produktionsflexibilität.

Auf technologischer Ebene ist eine zentrale Datenhaltung hilfreich, um flexibel auf Produktionsschwankungen im gesamten Netzwerk zu reagieren oder um durch präziseres Tracking von Produktionsschritten und Logistikdienstleistungen den Lieferservice zu verbessern.

Abschließend können wiederkehrende, transaktionsbezogene und Inseratgebühren genutzt werden, um Preise dynamischer zu gestalten. So können beispielweise Sonderaktionen durchgeführt werden, bei denen kurzzeitig die Gebühren gesenkt werden oder gänzlich wegfallen.

### Gestaltungsmöglichkeiten zur Erreichung technischer Ziele

Die technischen Ziele werden hauptsächlich von der Plattformtechnologie beeinflusst. Jedoch gibt es auch einige Gestaltungsmaßnahmen, die unerwartet technische Ziele beeinflussen. So kann etwa die geographische Reichweite die Zuverlässigkeit von Systemen negativ beeinträchtigen. Weltweite Kommunikationsnetze sind anfälliger für Störungen oder hohe Latenzzeiten. International unterschiedliche Standards und Dateiformate für verschiedene Produktionsverfahren können zudem die Interoperabilität negativ beeinflussen.

Während zusätzliche und komplexe Marktmechanismen die Nutzerfreundlichkeit verbessern können, sind komplizierte Algorithmen zur Preisfindung oder dem Matching schwerer wartbar. Sollten diese Verfahren auf künstlicher Intelligenz basieren, kann bei unvorhergesehenem Verhalten auf dem Marktplatz die Stabilität nicht unbedingt gewährleistet werden.

Auch Governance-Maßnahmen haben Auswirkungen auf technische Ziele. So können nicht nur Zertifizierungen für Qualitätsmanagement von der Plattform verlangt werden, sondern auch Zertifizierungen für die Einhaltung von Standards und Prozessen die Interoperabilität stark vereinfachen. Feedbackmechanismen erhöhen zwar die Nutzerfreundlichkeit, gleichzeitig müssen hier technische Maßnahmen implementiert und gewartet werden, die Missbrauch und Spam unterbinden.

### Gestaltungsmöglichkeiten zur Erreichung organisatorischer Ziele

Gestaltungselemente im Bereich Qualitätsmanagement beeinflussen einige organisatorische Ziele. So tragen umfangreiche Steuerungsmöglichkeiten durch eine enge Eingrenzung der Akteure, Prüfungen und Zertifizierungen der Akteure positiv zur Reduzierung von Störgrößen und damit der Erreichung des Ziels Governance bei. Langfristige Beziehungen bieten die Möglichkeit, striktere Regeln über einen längeren Zeitraum durchzusetzen, wohingegen kurzfristige Beziehungen ohne starre Rahmenverträge positiv zur Erreichung von Agilität beitragen. Insgesamt bietet wenig Funktionalität eine höhere Anpassungsfähigkeit. So schafft beispielsweise die Integration von Logistik- oder Zahlungsdienstleitern Abhängigkeiten, welche einen negativen Einfluss auf die Agilität haben.

Das Ziel Zugänglichkeit wird, entgegen dem Ziel Governance, durch eine hohe Offenheit gegenüber Plattformteilnehmern erreicht. Zur Zielerreichung sollte keine Einschränkung von Teilnehmern in Bezug auf Geografie oder Fertigungsverfahren vorgenommen, neutrale Onlinekatalogisierung bestehen, keine Qualitätssicherung oder Zertifizierungsmaßnahmen eingefordert sowie keine einmaligen Fixgebühren erhoben werden. Barrieren können entsprechend reduziert und ein schneller und einfacher Zugang zum Markt gewährleistet werden.

Eng mit diesen Gestaltungselementen verbunden kann Neutralität gewährleistet werden, indem keine Benutzergruppen ausgeschlossen werden. Wenn Eingrenzungen erfolgen, sollten diese für beide Parteien (Käufer und Verkäufer) gleichermaßen implementiert sein. Opportunistisches Verhalten und damit ein negativer Einfluss auf die Neutralität werden durch manuelle Eingriffe des Plattformbetreibers begünstigt, beispielsweise durch manuelles Matching, Schlichtungsmaßnahmen oder Feedback durch die Plattform. Ebenso sollten zur Erreichung von Neutralität die Gebührenmodelle für alle Teilnehmergruppen gleich sein.

### Gestaltungsmöglichkeiten zur Erreichung sozialer und ökologischer Ziele

Soziale und ökologische Ziele werden maßgeblich von dem Dienstleistungsumfang der Plattform beeinflusst. Transaktionsdienstleister verfügen im Vergleich zu Vermittlungsdienstleistern über mehr Handlungs- und Entscheidungsspielraum als Vermittlungsdienstleister. Ein größerer Dienstleistungsumfang wirkt sich entsprechend positiv auf Ziele wie Nutzerzufriedenheit, Vertrauen, Nachhaltigkeit und Reputation aus.

Die Eingrenzung der Teilnehmer anhand der geographischen Reichweite oder Fertigungsverfahren sowie langfristige Beziehungen schaffen zwar durch das Gefühl einer engen Betreuung und umfangreichen Zugangsmechanismen Vertrauen, beeinflussen jedoch negativ die Möglichkeit eines großen Bekanntheitsgrads und Erreichung des Ziels Reputation. Ergänzend hat der Plattformbetreiber Handlungsspielraum bezüglich der Wahl ökologisch nachhaltiger Fertigungsverfahren und -materialen, sollte eine Einschränkung der Verfahren vorgesehen sein. Erfolgt keine Einschränkung der Fertigungsverfahren, ist hingegen eine höhere Nutzerzufriedenheit durch ein breiteres Angebot zu erwarten. Die Nutzerzufriedenheit lässt sich insgesamt durch eine Funktionsvielfalt, wie Mehrwertdienste, Priorisierungsmöglichkeiten oder Integrationsmöglichkeiten externer Systeme erreichen.

Ferner haben Aktivitäten zu Qualitätsmanagement und Governance einen großen Einfluss auf die Erreichung dieser Zieldimension. Je mehr Prüfung und Kontrolle der Plattformbetreiber durchführt, desto höher ist das Vertrauen der Teilnehmer. Ökologische Nachhaltigkeit wird ferner durch umfangreiche Qualitätsprüfung erreicht, sodass eine wiederholte Fertigung aufgrund von Qualitätsfehlern vermieden werden kann. Das Ziel Nachhaltigkeit kann beispielsweise durch eine Eingrenzung der geographischen Eingrenzung und damit geringe Transportwege oder entsprechenden Verkäuferzertifizierungen positiv beeinflusst werden. Ferner lässt sich durch die Implementierung von Feedbackmechanismen durch den Plattformbetreiber und entsprechende umfangreiche und positive Feedbacks Reputation aufbauen. Die Zusammenarbeit mit verschiedenen Drittdienstleistern für Zahlungen oder Logistik und deren Integration in die Plattform kann ebenso einen positiven Einfluss auf die Reputation haben und ermöglicht eine Auswahl hinsichtlich ökologischer Aspekte wie regionale Nähe.

## Fazit

Die Kapazitätsauslastung von Produktionsanlagen und -maschinen sowie der Umgang mit Kapazitätsauslastungsrisiken ist ein wesentlicher Erfolgsfaktor produzierender Unternehmen. Doch die Unsicherheit, die sich aus der zukünftigen Nachfrage oder der Verfügbarkeit von Maschinen ergibt, stellt in diesem Zusammenhang eine große Herausforderung dar. Die Sharing Economy revolutioniert durch die Möglichkeit eines Handels von Fertigungskapazitäten bereits seit einigen Jahren traditionelle Organisationsstrukturen zur interorganisatorischen Zusammenarbeit in Produktionsnetzwerken. Dabei bieten digitale Plattformen eine geeignete Infrastruktur zur Vermittlung von Anbietern und Nachfragern von Fertigungskapazitäten sowie der Abwicklung kompletter Handelstransaktionen.

In der Literatur finden sich nur wenige Publikationen, die sich mit Plattformen für Produktionsnetzwerke auseinandersetzen. Insbesondere bleiben in den bestehenden Veröffentlichungen die Ziele dieser spezifischen Plattformart unberücksichtigt. Aufbauend auf dieser Forschungslücke konnte diese Arbeit durch einen stufenweisen Prozess basierend auf einer Literaturanalyse drei Forschungsziele (FZ) erreichen:

### FZ1

Analyse von Zieldimensionen und Zielen digitale Plattformen im Allgemeinen sowie die Übertragung geeigneter Ziele auf digitale Plattformen für Produktionsnetzwerke

### FZ2

Ergänzung von bisher nicht berücksichtigten Zielen digitaler Plattformen für Produktionsnetzwerke und Entwicklung eines gesamtheitlichen Zielmodells

### FZ3

Ableitung von Empfehlungen für das Design digitaler Plattformen für Produktionsnetzwerke zur Zielerreichung

In FZ1 wurden verschiedene Zielmodelle mit unterschiedlichen Dimensionen und Zielen identifiziert und miteinander verglichen. Davon erwiesen sich die fünf Zieldimensionen *wirtschaftliche Ziele, Marktleistungsziele, technische Ziele, organisatorische Ziele* sowie *soziale & ökologische Ziele* mit insgesamt 21 Zielen als geeignet für den Untersuchungsgegenstand der vorliegenden Arbeit. Eine Konzeptmatrix diente der strukturierten Identifizierung und Strukturierung der Ziele. Im Rahmen von FZ2 wurde die bisher nicht betrachtete Dimension *produktionsbezogene Marktleistungsziele* mit entsprechenden Zielen ergänzt und damit das Zielmodell auf digitale Plattformen für Produktionsnetzwerke angepasst. Das finale Zielmodell mit sechs Dimensionen und 25 Zielen wurde in Abschn. 4 vorgestellt. Das Modell leistet einen wesentlichen Beitrag zur Reduktion der Komplexität von Plattformzielen. Ferner fungiert die Zielübersicht als Steuerungsinstrument zur kontinuierlichen Überwachung der Leistungsfähigkeit der Plattform. Dazu wurde die Anwendung des Zielmodells beispielhaft in FZ3 präsentiert. Die Analyse des Einflusses bestimmter Gestaltungselemente auf die Zielerreichung ermöglicht einige Design-Empfehlungen.

Neben den erreichten Zielen weist die vorliegende Untersuchung einige Limitationen auf. Die Ziele, beispielsweise Wachstum oder Nachhaltigkeit wurden sehr eng definiert, sodass Empfehlungen zur Zielerreichung ebenso spezifischer formuliert und damit Ziele erreicht werden können. Damit werden einige Aspekte der Ziele ausgeschlossen. Ferner sind für die Entwicklung von Gestaltungsempfehlungen nicht nur definierte Ziele, sondern auch Gestaltungsmerkmale, d. h. Strukturelemente notwendig. Diese Arbeit stützt sich auf eine Auswahl von Strukturelementen aus der Literatur, welche in Abschn. 2 zusammengefasst wurden. Ferner basieren die Gestaltungsempfehlungen auf den Erkenntnissen der Strukturelemente in Abschn. 2 und dem Zielmodell in Abschn. 4 und wurden durch Expertenworkshops erarbeitet. Entsprechend wurde sich auf eine Auswahl von Empfehlungen konzentriert, zu welchen Einigkeit in der Forschungsgruppe bestand. Das Kapitel bietet damit eine geeignete Grundlage für weitere Forschung. Zur Entwicklung eines gesamtheitlichen Struktur-Ziel-Einflussmodells ließe sich in nächsten Forschungsschritten zunächst ein umfängliches und auf digitale Plattformen für Produktionsnetzwerke angepasstes Strukturmodell erarbeiten. Die Erarbeitung einer Taxonomie wäre geeignet, sodass sowohl konzeptionelle als auch empirische Iterationsschritte durchgeführt werden können. Weiterhin empfiehlt sich die detaillierte Analyse der Zielbeeinflussung untereinander sowie die Gewichtung der Bedeutung spezifischer Ziele. Ferner sollten die Gestaltungsmöglichkeiten zur Zielerreichung beispielsweise anhand von Fallstudien evaluiert werden.

## Supplementary Information

Onlinematerial 1: Konzeptmatrix zu Strukturelementen digitaler Plattformen für Produktionsnetzwerke

Onlinematerial 2: Konzeptmatrix zur Entwicklung des Zielmodells
