# Supplementary material for: Goals and design of digital platforms for production networks
Source: HMD Prax Wirtsch Inform. 2022 Sep 6;59(5):1281–311. [Article in German] doi: 10.1365/s40702-022-00908-2 (PMC9446587; doi:10.1365/s40702-022-00908-2)
Supplement: Supplementary file 1 — Onlinematerial 1: Konzeptmatrix zu Strukturelementen digitaler Plattformen für Produktionsnetzwerke [file 40702_2022_908_MOESM1_ESM.pdf]

Onlinematerial 1: Konzeptmatrix zu Strukturelementen digitaler Plattformen für Produktionsnetzwerke

	Dienstleistungsumfang	Akteure	Marktmechanismen	Qualitätsmanagement & Governance	Plattformtechnologie	Einnahmequellen
Afuah (2013)	•					
Albrecht et al. (2005)					•	
Alt et al. (2002)		•	•		•	•
Aulkemeier et al. (2019)					•	
Bailey & Bakos (1997)	•		•			
Bakos (1998)			•		•	
Banker et al. (2006)					•	
Beam & Segev (1997)		•				
Bhargava & Choudhary (2004)	•					
Björkdahl (2020)					•	
Blaschke et al. (2019)	•	•		•	•	
Broekhuizen et al. (2021)		•				
Camarinha-Matos (2009)				•		
Chaudhuri et al. (2021)						•
Choi & Mela (2019)						•
Cusumano (2010)					•	
Dai & Kauffman (2002)	•		•		•	
Datta & Chatterjee (2008)	•					
De Reuver et al. (2018)		•		•	•	
Dou & Chou (2002)	•				•	
Duan et al. (2019)		•	•			
Eisenmann et al. (2009)		•				•
Essig & Arnold (2001)		•				
Evans (2003)						•
Fairchild et al. (2004)				•	•	
Freitag et al. (2020)						•
Galbreth et al. (2005)						•
Gallien & Wein (2005)			•			
Ghazawneh & Henfridsson (2015)		•		•	•	
Grewal et al. (2010)		•		•	•	
Grieger (2003)		•				
Gunasekaran et al. (2002)				•	•	
Gupta et al. (2005)		•		•		
Helo et al. (2019)			•		•	
Howard et al. (2006)						•
Janita & Miranda (2013)	•				•	
Kalvenes & Basu (2006)		•		•	•	
Kauffman et al. (2010)					•	
Kazan et al. (2018)		•		•	•	
Kim (2016)				•		•
Kim & Ahn (2007)				•		
Lancastre & Lages (2006)		•				
Lennstrand et al. (2001)			•			
Li et al. (2010)						•
Lu & Antony (2003)		•	•			
Lu et al. (2016)			•			
Mahalingam et al. (2020)				•		
Malone et al. (1987)						•
Mishra et al. (2016)					•	
Mofidi & Pazour (2019)			•			
Movahedi et al. (2012)		•	•			
Murtaza et al. (2004)					•	
O'Reilly & Finnegan (2010)	•	•				
Ondrus et al. (2015)		•			•	
Oppong-Tawiah et al. (2020)	•					
Ordanini et al. (2004)						•
Pavlou & Gefen (2004)				•		
Perscheid et al. (2020)					•	•
Petersen et al. (2007)	•					
Pressey & Ashton (2009)		•				
Reimers (1996)				•		•
Schreieck et al. (2017)		•		•		
Serel (2007)		•	•			
Sharifi et al. (2006)	•	•				
Smits & Weigand (2010)	•	•	•	•		
Soh & Markus (2002)	•	•				
Spulber (2019)	•	•				
Standing et al. (2006)		•		•		
Standing & Standing (2015)	•					
Standing et al. (2010)						•
Stein et al. (2019)			•			
Strader & Shaw (1997)					•	
Sun (2010)					•	
Täuscher & Laudien (2018)	•	•	•	•	•	•
Teo et al. (2006)					•	
Tiwana et al. (2010)		•		•		
Thitimajshima et al. (2018)	•				•	
Tsatsou et al. (2010)				•		
Tura et al. (2018)				•		
Vakeel et al. (2020)		•	•			
Wang & Benaroch (2004)	•	•				•
Wang & Nandhakumar (2017)		•				
Wang et al. (2008)				•		
Wang et al. (2018)				•		
Wang et al. (2019)					•	
White et al. (2007)		•				
Wulf & Blohm (2020)					•	
Yao & Zhu (2012)					•	
Yoo et al. (2007)		•	•			
Yu & Ramaprasad (2019)						•
Zheng (2006)						•
Zutshi & Grilo (2019)				•		

Literaturverzeichnis zu Onlinematerial 1

Afuah A. (2013): Are network effects really all about size? The role of structure and conduct. *Strategic Management Journal*: 34(3), S. 257–273. DOI: 10.1002/smj.2013.

Albrecht C.C., Dean D.L., Hansen J. V. (2005): Marketplace and technology standards for B2B e-commerce: progress, challenges, and the state of the art. *Information and Management*: 42(6), S. 865–875. DOI: 10.1016/j.im.2004.09.003.

Alt R., Cäsar M.A., Grau J.U. (2002): Collaboration in the consumer product goods industry - Analysis of marketplaces. *10th European Conference on Information Systems*(92), S. 582–595.

Aulkemeier F., Iacob M.E., van Hillegersberg J. (2019): Platform-based collaboration in digital ecosystems. *Electronic Markets*: 29(4), S. 597–608. DOI: 10.1007/s12525-019-00341-2.

Bailey J.P., Bakos J.Y. (1997): An Exploratory Study of the Emerging Role of Electronic Intermediaries. *International Journal of Electronic Commerce*: 1(3), S. 7–20. DOI: 10.1080/10864415.1997.11518287.

Bakos J.Y. (1998): The emerging role of electronic marketplaces on the internet. *Communications of the ACM*: 41(8), S. 35–42. DOI: 10.1145/280324.280330.

Banker R.D., Bardhan I.R., Chang H., Lin S. (2006): Plant information systems, manufacturing capabilities, and plant performance. *Management Information Systems Quarterly*: 30(2), S. 315–337.

Beam C., Segev A. (1997): Automated negotiations: A survey of the state of the art. *Wirtschaftsinformatik*: 39(3), S. 263–268.

Bhargava H.K., Choudhary V. (2004): Economics of an information intermediary with aggregation benefits. *Information Systems Research*: 15(1), S. 22–36. DOI: 10.1287/isre.1040.0014.

Björkdahl J. (2020): Strategies for digitalization in manufacturing firms. *California Management Review*: 62(4), S. 17–36. DOI: 10.1177/0008125620920349.

Blaschke M., Haki K., Aier S., Winter R. (2019): Taxonomy of Digital Platforms: A Platform Architecture Perspective. *Proceedings of the International Conference on Wirtschaftsinformatik*, S. 572–586.

Broekhuizen T.L.J., Emrich O., Gijsenberg M.J., Broekhuis M., Donkers B., Sloot L.M. (2021): Digital platform openness: Drivers, dimensions and outcomes. *Journal of Business Research*: 122(o. A.), S. 902–914. DOI: 10.1016/j.jbusres.2019.07.001.

Camarinha-Matos L.M., Afsarmanesh H., Galeano N., Molina A. (2009): Collaborative networked organizations - Concepts and practice in manufacturing enterprises. *Computers and Industrial Engineering*: 57(1), S. 46–60. DOI: 10.1016/j.cie.2008.11.024.

Chaudhuri A., Datta P.P., Fernandes K.J., Xiong Y. (2021): Optimal pricing strategies for manufacturing-as-a service platforms to ensure business sustainability. *International Journal of Production Economics*: 234(o. A.), S. 1–26. DOI: 10.1016/j.ijpe.2021.108065.

Choi H., Mela C.F. (2019): Monetizing online marketplaces. *Marketing Science*: 38(6), S. 948–972. DOI: 10.1287/mksc.2019.1197.

Cusumano M. (2010): Cloud computing and SaaS as new computing platforms. *Communications of the ACM*: 53(4), S. 27–29. DOI: 10.1145/1721654.1721667.

Dai Q., Kauffman R.J. (2002): Business Models for Internet-Based B2B Electronic Markets. *International Journal of Electronic Commerce*: 6(4), S. 41–72. DOI: 10.1080/10864415.2002.11044247.

Datta P., Chatterjee S. (2008): The economics and psychology of consumer trust in intermediaries in electronic markets: The EM-trust framework. *European Journal of Information Systems*: 17(1), S. 12–28. DOI: 10.1057/palgrave.ejis.3000729.

Dou W., Chou D.C. (2002): A structural analysis of business-to-business digital markets. *Industrial Marketing Management*: 31(2), S. 165–176. DOI: 10.1016/S0019-8501(01)00177-8.

Duan S.X., Deng H., Luo F. (2019): An integrated approach for identifying the efficiency-oriented drivers of electronic markets in electronic business. *Journal of Enterprise Information Management*: 32(1), S. 60–74. DOI: 10.1108/JEIM-05-2018-0090.

Eisenmann T.R., Parker G., van Alstyne M.W. (2009): Opening platforms: How, when and why? In: Gawer A. (Hrsg.): *Platforms, Markets and Innovation*, S. 131–162. Cheltenham, U.K.

Essig M., Arnold U. (2001): Electronic procurement in supply chain management: An information economics-based analysis of electronic markets. *Journal of Supply Chain Management*: 37(3), S. 43–49. DOI: 10.1111/j.1745-493X.2001.tb00112.x.

Evans D.S. (2003): Some Empirical Aspects of Multi-sided Platform Industries. *Review of Network Economics*: 2(3), S. 191–209. DOI: 10.2202/1446-9022.1026.

Fairchild A.M., Ribbers P.M.A., Nooteboom A.O. (2004): A success factor model for electronic markets: Defining outcomes based on stakeholder context and business process. *Business Process Management Journal*: 10(1), S. 63–79. DOI: 10.1108/14637150410518338.

Freitag B., Häfner L., Pfeuffer V., Übelhör J. (2020): Evaluating investments in flexible on-demand production capacity: a real options approach. *Business Research*: 13(1), S. 133–161. DOI: 10.1007/s40685-019-00105-w.

Galbreth M.R., March S.T., Scudder G.D., Shor M. (2005): A game-theoretic model of e-marketplace participation growth. *Journal of Management Information Systems*: 22(1), S. 295–319. DOI: 10.1080/07421222.2003.11045837.

Gallien J., Wein L.M. (2005): A Smart Market for Industrial Procurement with Capacity Constraints. *Management Science*: 51(1), S. 76–91. DOI: 10.1287/mnsc.1040.0230.

Ghazawneh A., Henfridsson O. (2015): A paradigmatic analysis of digital application marketplaces. *Journal of Information Technology*: 30(3), S. 198–208. DOI: 10.1057/jit.2015.16.

Grewal R., Chakravarty A., Saini A. (2010): Governance mechanisms in business-to-business electronic markets. *Journal of Marketing*: 74(4), S. 45–62. DOI: 10.1509/jmkg.74.4.45.

Grieger M. (2003): Electronic marketplaces: A literature review and a call for supply chain management research. *European Journal of Operational Research*: 144(2), S. 280–294. DOI: 10.1016/S0377-2217(02)00394-6.

Gunasekaran A., Marri H.B., McGaughey R.E., Nebhwani M.D. (2002): E-commerce and its impact on operations management. *International Journal of Production Economics*: 75(1–2), S. 185–197. DOI: 10.1016/S0925-5273(01)00191-8.

Gupta S., Cadeaux J., Woodside A. (2005): Mapping network champion behavior in B2B electronic venturing. *Industrial Marketing Management*: 34(5), S. 495–503. DOI: 10.1016/j.indmarman.2004.10.006.

Helo P., Phuong D., Hao Y. (2019): Cloud manufacturing – Scheduling as a service for sheet metal manufacturing. *Computers and Operations Research*: 110(o. A.), S. 1–31. DOI: 10.1016/j.cor.2018.06.002.

Howard M., Vidgen R., Powell P. (2006): Automotive e-hubs: Exploring motivations and barriers to collaboration and interaction. *The Journal of Strategic Information Systems*: 15(1), S. 51–75. DOI: 10.1016/j.jsis.2005.06.002.

Janita M.S., Miranda F.J. (2013): The antecedents of client loyalty in business-to-business (B2B) electronic marketplaces. *Industrial Marketing Management*: 42(5), S. 814–823. DOI: 10.1016/j.indmarman.2013.01.006.

Kalvenes J., Basu A. (2006): Design of robust business-to-business electronic marketplaces with guaranteed privacy. *Management Science*: 52(11), S. 1721–1736. DOI: 10.1287/mnsc.1060.0570.

Kauffman R.J., Li T., Van Heck E. (2010): Business network-based value creation in electronic commerce. *International Journal of Electronic Commerce*: 15(1), S. 113–144. DOI: 10.2753/JEC1086-4415150105.

Kazan E., Tan C.W., Lim E.T.K., Sørensen C., Damsgaard J. (2018): Disentangling Digital Platform Competition: The Case of UK Mobile Payment Platforms. *Journal of Management Information Systems*: 35(1), S. 180–219. DOI: 10.1080/07421222.2018.1440772.

Kim J. (2016): The platform business model and business ecosystem: quality management and revenue structures. *European Planning Studies*: 24(12), S. 2113–2132. DOI: 10.1080/09654313.2016.1251882.

Kim M.S., Ahn J.H. (2007): Management of trust in the e-marketplace: The role of the buyer's experience in building trust. *Journal of Information Technology*: 22(2), S. 119–132. DOI: 10.1057/palgrave.jit.2000095.

Lancastre A., Lages L.F. (2006): The relationship between buyer and a B2B e-marketplace: Cooperation determinants in an electronic market context. *Industrial Marketing Management*: 35(6), S. 774–789. DOI: 10.1016/j.indmarman.2005.03.011.

Lennstrand B., Frey M., Johansen M. (2001): Analyzing B2B eMarkets -the Impact of Product and Industry Characteristics on Value Creation and Business Strategies. *ITS ASia-Indian Ocean Regional Conference in Perth*: 1(1), S. 1–22.

Li S., Liu Y., Bandyopadhyay S. (2010): Network effects in online two-sided market platforms: A research note. *Decision Support Systems*: 49(2), S. 245–249. DOI: 10.1016/j.dss.2010.02.004.

Lu D., Antony F. (2003): Implications of B2B marketplace to supply chain development. *TQM Magazine*: 15(3), S. 173–179. DOI: 10.1108/09544780310469271.

Lu Y., Gupta A., Ketter W., van Heck E. (2016): Exploring bidder heterogeneity in multichannel sequential B2B auctions. *Management Information Systems Quarterly*: 40(3), S. 645–662. DOI: 10.25300/MISQ/2016/40.3.06.

Mahalingam A., Alyakoob M., Rahman M. (2020): Implications of priority access in markets with experts: Evidence from online marketplace lending. *41st International Conference on Information Systems*, S. 1–17.

Malone T.W., Yates J., Benjamin R.I. (1987): Electronic Markets and Electronic Hierarchies. *Communications of the ACM*: 30(6), S. 484–497.

Mishra N., Singh A., Kumari S., Govindan K., Ali S.I. (2016): Cloud-based multi-agent architecture for effective planning and scheduling of distributed manufacturing. *International Journal of Production Research*: 54(23), S. 7115–7128. DOI: 10.1080/00207543.2016.1165359.

Mofidi S.S., Pazour J.A. (2019): When is it beneficial to provide freelance suppliers with choice? A hierarchical approach for peer-to-peer logistics platforms. *Transportation Research Part B: Methodological*: 126(o. A.), S. 1–23. DOI: 10.1016/j.trb.2019.05.008.

Movahedi B., Lavassani K.M., Kumar V. (2012): E-Marketplace emergence: Evolution, developments and classification. *Journal of Electronic Commerce in Organizations*: 10(1), S. 14–32. DOI: 10.4018/jeco.2012010102.

Murtaza M.B., Gupta V., Carroll R.C. (2004): E-marketplaces and the future of supply chain management: Opportunities and

challenges. *Business Process Management Journal*: 10(3), S. 325–335. DOI: 10.1108/14637150410539722.

O'Reilly P., Finnegan P. (2010): Intermediaries in inter-organisational networks: Building a theory of electronic marketplace performance. *European Journal of Information Systems*: 19(4), S. 462–480. DOI: 10.1057/ejis.2010.12.

Ondrus J., Gannamaneni A., Lyytinen K. (2015): The impact of openness on the market potential of multi-sided platforms: A case study of mobile payment platforms. *Journal of Information Technology*: 30(3), S. 260–275. DOI: 10.1057/jit.2015.7.

Oppong-Tawiah D., Bassellier G., Pinsonneault A. (2020): Tracing the next-generation platform firm: A typology of digital platforms as new organizing forms. *28th European Conference on Information Systems*, S. 1–10.

Ordanini A., Micelli S., Di Maria E. (2004): Failure and success of B-to-B exchange business models: A contingent analysis of their performance. *European Management Journal*: 22(3), S. 281–289. DOI: 10.1016/j.emj.2004.04.013.

Pavlou P.A., Gefen D. (2004): Building effective online marketplaces with institution-based trust. *Information Systems Research*: 15(1), S. 37–59. DOI: 10.1287/isre.1040.0015.

Perscheid G., Ostern N.K., Moormann J. (2020): Towards a taxonomy of decentralized platform-based business models. *28th European Conference on Information Systems*, S. 1–16.

Petersen K.J., Ogden J.A., Carter P.L. (2007): B2B e-marketplaces: A typology by functionality. *International Journal of Physical Distribution and Logistics Management*: 37(1), S. 4–18. DOI: 10.1108/09600030710723291.

Pressey A.D., Ashton J.K. (2009): The antitrust implications of electronic business-to-business marketplaces. *Industrial Marketing Management*: 38(4), S. 468–476. DOI: 10.1016/j.indmarman.2008.02.012.

Reimers K. (1996): The non-market preconditions of electronic markets: Implications for their evolution and applicability. *European Journal of Information Systems*: 5(2), S. 75–83. DOI: 10.1057/ejis.1996.14.

De Reuver M., Sørensen C., Basole R.C. (2018): The digital platform: A research agenda. *Journal of Information Technology*: 33(2), S. 124–135. DOI: 10.1057/s41265-016-0033-3.

Schreieck M., Wiesche M., Krcmar H. (2017): The platform owner' s challenge to capture value-insights from a business-to-business IT platform. *38th International Conference on Information Systems*, S. 1–19.

Serel D.A. (2007): Capacity reservation under supply uncertainty. *Computers and Operations Research*: 34(4), S. 1192–1220. DOI: 10.1016/j.cor.2005.06.018.

Sharifi H., Kehoe D.F., Hopkins J. (2006): A classification and selection model of e-marketplaces for better alignment of supply chains. *Journal of Enterprise Information Management*: 19(5), S. 483–503. DOI: 10.1108/17410390610703639.

Smits M., Weigand H. (2010): Identifying market performance indicators that can be influenced by electronic intermediaries. *18th European Conference on Information Systems*, S. 1–13.

Soh C., Markus M.L. (2002): Business-to-business e-marketplaces: A strategic archetypes approach. *23rd International Conference on Information Systems*, S. 12–31.

Spulber D.F. (2019): The economics of markets and platforms. *Journal of Economics and Management Strategy*: 28(1), S. 159–172. DOI: 10.1111/jems.12290.

Standing C., Love P.E.D., Stockdale R., Gengatharen D. (2006): Examining the relationship between electronic marketplace strategy and structure. *IEEE Transactions on Engineering Management*: 53(2), S. 297–311. DOI: 10.1109/TEM.2005.861801.

Standing S., Standing C. (2015): Service value exchange in B2B electronic marketplaces. *Journal of Business and Industrial Marketing*: 30(6), S. 723–732. DOI: 10.1108/JBIM-05-2014-0112.

Standing S., Standing C., Love P.E.D. (2010): A review of research on e-marketplaces 1997–2008. *Decision Support Systems*: 49(1), S. 41–51. DOI: 10.1016/j.dss.2009.12.008.

Stein N., Walter B., Flath C.M. (2019): Towards open production: Designing a marketplace for 3D-printing capacities. *40th International Conference on Information Systems*, S. 1–15.

Strader T.J., Shaw M.J. (1997): Characteristics of electronic markets. *Decision Support Systems*: 21(3), S. 185–198. DOI: 10.1016/S0167-9236(97)00028-6.

Sun H. (2010): Sellers' trust and continued use of online marketplaces. *Journal of the Association for Information Systems*: 11(4), S. 182–211. DOI: 10.17705/1jais.00226.

Täuscher K., Laudien S.M. (2018): Understanding platform business models: A mixed methods study of marketplaces. *European Management Journal*: 36(3), S. 319–329. DOI: 10.1016/j.emj.2017.06.005.

Teo T.S.H., Ranganathan C., Dhaliwal J. (2006): Key dimensions of inhibitors for the deployment of web-based business-to-business electronic commerce. *IEEE Transactions on Engineering Management*: 53(3), S. 395–411. DOI: 10.1109/TEM.2006.878106.

Thitimajshima W., Esichaikul V., Krairit D. (2018): A framework to identify factors affecting the performance of third-party B2B e-marketplaces: A seller's perspective. *Electronic Markets*: 28(2), S. 129–147. DOI: 10.1007/s12525-017-0256-3.

Tiwana A., Konsynski B., Bush A.A. (2010): Platform evolution: Coevolution of platform architecture, governance, and

environmental dynamics. *Information Systems Research*: 21(4), S. 675–687. DOI: 10.1287/isre.1100.0323.

Tsatsou P., Elaluf-Calderwood S., Liebenau J. (2010): Towards a taxonomy for regulatory issues in a digital business ecosystem in the EU. *Journal of Information Technology*: 25(3), S. 288–307. DOI: 10.1057/jit.2009.22.

Tura N., Kutvonen A., Ritala P. (2018): Platform design framework: conceptualisation and application. *Technology Analysis and Strategic Management*: 30(8), S. 881–894. DOI: 10.1080/09537325.2017.1390220.

Vakeel K.A., Malthouse E.C., Yang A. (2020): Impact of network effects on service provider performance in digital business platforms. *Journal of Service Management*: 32(4), S. 461–482. DOI: 10.1108/JOSM-04-2020-0120.

Wang C.X., Benaroch M. (2004): Supply chain coordination in buyer centric B2B electronic markets. *International Journal of Production Economics*: 92(2), S. 113–124. DOI: 10.1016/j.ijpe.2003.09.016.

Wang Y., Lin Y., Zhong R.Y., Xu X. (2019): IoT-enabled cloud-based additive manufacturing platform to support rapid product development. *International Journal of Production Research*: 57(12), S. 3975–3991. DOI: 10.1080/00207543.2018.1516905.

Wang G., Nandhakumar J. (2017): Strategic swaying: How startups grow digital platforms. *38th International Conference on Information Systems*, S. 1–17.

Wang Y., Qu Z., Tan B. (2018): How do assurance mechanisms interact in online marketplaces? A signaling perspective. *IEEE Transactions on Engineering Management*: 65(2), S. 239–251. DOI: 10.1109/TEM.2017.2786275.

Wang S., Zheng S., Xu L., Li D., Meng H. (2008): A literature review of electronic marketplace research: Themes, theories and an integrative framework. *Information Systems Frontiers*: 10(5), S. 555–571. DOI: 10.1007/s10796-008-9115-2.

White A., Daniel E., Ward J., Wilson H. (2007): The adoption of consortium B2B e-marketplaces: An exploratory study. *Journal of Strategic Information Systems*: 16(1), S. 71–103. DOI: 10.1016/j.jsis.2007.01.004.

Wulf J., Blohm I. (2020): Fostering value creation with digital platforms: A unified theory of the application programming interface design. *Journal of Management Information Systems*: 37(1), S. 251–281. DOI: 10.1080/07421222.2019.1705514.

Yao Y., Zhu K.X. (2012): Do electronic linkages reduce the bullwhip effect? An empirical analysis of the U.S. manufacturing supply chains. *Information Systems Research*: 23(3-part-2), S. 1042–1055. DOI: 10.1287/isre.1110.0394.

Yoo B., Choudhary V., Mukhopadhyay T. (2007): Electronic B2B marketplaces with different ownership structures. *Management Science*: 53(6), S. 952–961. DOI: 10.1287/mnsc.1060.0685.

Yu Y., Ramaprasad J. (2019): Engagement on digital platforms : A theoretical perspective. *40th International Conference on Information Systems*(40), S. 1–8.

Zheng W. (2006): The Business Models of E-Marketplace. *Communications of the IIMA*: 6(4), S. 1–18.

Zutshi A., Grilo A. (2019): The Emergence of Digital Platforms: A Conceptual Platform Architecture and impact on Industrial Engineering. *Computers and Industrial Engineering*: 136(November), S. 546–555. DOI: 10.1016/j.cie.2019.07.027.
