# Supplementary material for: Goals and design of digital platforms for production networks
Source: HMD Prax Wirtsch Inform. 2022 Sep 6;59(5):1281–311. [Article in German] doi: 10.1365/s40702-022-00908-2 (PMC9446587; doi:10.1365/s40702-022-00908-2)
Supplement: Supplementary file 2 — Onlinematerial 2: Konzeptmatrix zur Entwicklung des Zielmodells [file 40702_2022_908_MOESM2_ESM.pdf]

Onlinematerial 2: Konzeptmatrix zur Entwicklung des Zielmodells

	Wirtschaftliche Ziele					Marktleistungsziele				Technische Ziele				Organisatorische Ziele				Soziale & ökologische Ziele			
**Artikel**	Gewinn	Wachstum	Nutzeranzahl	Transaktionsanzahl, -vol.	Marktanteil	Nutzerservice	Nutzertreue	Sicherheit & Datenschutz	Transaktionsservice	Nutzerfreundlichkeit	Zuverlässigkeit	Hard- & Softwarequalität	Interoperabilität	Governance	Zugänglichkeit	Agilität	Neutralität	Vertrauen	Nachhaltigkeit	Reputation	Nutzerzufriedenheit
Alt & Klein (2011)			•	•				•						•							
Asadullah et al. (2018)		•											•	•					•		
Bai & Velamuri (2021)									•					•							
Bakos (1991)				•		•	•		•												
Bakos (1998)	•								•					•							
Blaschke & Brosius (2018)													•		•	•					
Böhme & Koble (2007)								•													
Broekhuizen et al. (2021)															•						
Bunduchi (2008)									•									•			
Chen et al. (2016)											•										
Croitor & Adam (2020)														•							
Dai & Kauffman (2002)										•			•								
Datta & Chatterjee (2008)																		•			
De Reuver et al. (2018)													•	•	•	•					
Dou & Chou (2002)			•																		
Easton & Araujo (2003)				•		•					•	•									
Grewal et al. (2010)					•									•							
Gunasekaran et al. (2002)				•	•			•													
Hawlitschek et al. (2016)																		•			
Howard et al. (2006)														•	•		•				
Janita & Miranda (2013)							•	•													•
Kajan & Stoimenov (2005)													•								
Kalvenes & Basu (2006)								•													
Kim & Ahn (2007)																		•		•	
Mahadevan (2003)																	•				
Mahalingam et al. (2020)														•							
Mallapragada et al. (2015)																					•
Matook (2013)	•	•		•		•	•		•	•		•			•	•			•	•	•
Möhlmann (2021)																		•			
Ondrus et al. (2015)			•		•					•			•								
Oppong-Tawiah et al. (2020)		•											•								
Ordanini et al. (2004)			•	•									•				•				
Pavlou & Gefen (2004)							•											•			
Petersen et al. (2007)			•			•													•		
Pressey & Ashton (2009)			•														•				
Rolland et al. (2018)												•									
Saadatmand et al. (2017)													•								
Smits & Weigand (2010)				•					•			•			•		•				
Standing & Lin (2007)						•						•									
Standing et al. (2006)											•	•			•	•		•			•
Standing et al. (2010)														•						•	
Suh & Houston (2010)																		•		•	
Sun (2010)																		•			
Thitimajshima et al. (2018)			•		•	•	•	•	•	•		•						•		•	
Tuma (1998)																•					
Veisdal (2020)			•																		
Wang et al. (2008)														•							
Wang et al. (2011)						•										•					•
Wang et al. (2018)							•	•												•	
Wang et al. (2019)											•									•	•
Williams et al. (2008)	•	•				•				•		•				•					
Yoo et al. (2007)													•				•				
Alt & Klein (2011)						•									•						

Literaturverzeichnis zu Onlinematerial 2

Alt R., Klein S. (2011): Twenty years of electronic markets research—looking backwards towards the future. *Electronic Markets*: 21(1), S. 41–51. DOI: 10.1007/s12525-011-0057-z.

Asadullah A., Faik I., Kankanhalli A. (2018): Digital platforms: A review and future directions. *Proceedings of the 22nd Pacific Asia Conference on Information Systems - Opportunities and Challenges for the Digitized Society: Are We Ready?, PACIS 2018*, S. 1–14.

Bai G., Velamuri S.R. (2021): Contextualizing the sharing economy. *Journal of Management Studies*: 58(4), S. 977–1001. DOI: 10.1111/joms.12652.

Bakos J.Y. (1991): A strategic analysis of electronic marketplaces. *Management Information Systems Quarterly*: 15(3), S. 295–310. DOI: 10.2307/249641.

Bakos J.Y. (1998): The emerging role of electronic marketplaces on the internet. *Communications of the ACM*: 41(8), S. 35–42. DOI: 10.1145/280324.280330.

Blaschke M., Brosius M. (2018): Digital Platforms: Balancing Control and Generativity. *39th International Conference on Information Systems*, S. 1–9.

Böhme R., Koble S. (2007): Pricing strategies in electronic marketplaces with privacy-enhancing technologies. *Wirtschaftsinformatik*: 49(1), S. 16–25. DOI: 10.1007/s11576-007-0004-y.

Broekhuizen T.L.J., Emrich O., Gijsenberg M.J., Broekhuis M., Donkers B., Sloot L.M. (2021): Digital platform openness: Drivers, dimensions and outcomes. *Journal of Business Research*: 122(o. A.), S. 902–914. DOI: 10.1016/j.jbusres.2019.07.001.

Bunduchi R. (2008): Trust, power and transaction costs in B2B exchanges - A socio-economic approach. *Industrial Marketing Management*: 37(5), S. 610–622. DOI: 10.1016/j.indmarman.2007.05.003.

Chen F., Dou R., Li M., Wu H. (2016): A flexible QoS-aware web service composition method by multi-objective optimization in cloud manufacturing. *Computers and Industrial Engineering*: 99(o. A.), S. 423–431. DOI: 10.1016/j.cie.2015.12.018.

Croitor E., Adam M. (2020): Perceived input control on digital platforms: An empirical investigation. *28th European Conference on Information Systems*: o. A.(May), S. 1–17.

Dai Q., Kauffman R.J. (2002): Business Models for Internet-Based B2B Electronic Markets. *International Journal of Electronic Commerce*: 6(4), S. 41–72. DOI: 10.1080/10864415.2002.11044247.

Datta P., Chatterjee S. (2008): The economics and psychology of consumer trust in intermediaries in electronic markets: The EM-trust framework. *European Journal of Information Systems*: 17(1), S. 12–28. DOI: 10.1057/palgrave.ejis.3000729.

Dou W., Chou D.C. (2002): A structural analysis of business-to-business digital markets. *Industrial Marketing Management*: 31(2), S. 165–176. DOI: 10.1016/S0019-8501(01)00177-8.

Easton G., Araujo L. (2003): Evaluating the impact of B2B e-commerce: A contingent approach. *Industrial Marketing Management*: 32(5), S. 431–439. DOI: 10.1016/S0019-8501(03)00016-6.

Grewal R., Chakravarty A., Saini A. (2010): Governance mechanisms in business-to-business electronic markets. *Journal of Marketing*: 74(4), S. 45–62. DOI: 10.1509/jmkg.74.4.45.

Gunasekaran A., Marri H.B., McGaughey R.E., Nebhwani M.D. (2002): E-commerce and its impact on operations management. *International Journal of Production Economics*: 75(1–2), S. 185–197. DOI: 10.1016/S0925-5273(01)00191-8.

Hawlitschek F., Teubner T., Adam M.T.P.P., Borchers N.S., Möhlmann M., Weinhardt C. (2016): Trust in the sharing economy: An experimental framework. *37th International Conference on Information Systems*, S. 1–14.

Howard M., Vidgen R., Powell P. (2006): Automotive e-hubs: Exploring motivations and barriers to collaboration and interaction. *The Journal of Strategic Information Systems*: 15(1), S. 51–75. DOI: 10.1016/j.jsis.2005.06.002.

Janita M.S., Miranda F.J. (2013): The antecedents of client loyalty in business-to-business (B2B) electronic marketplaces. *Industrial Marketing Management*: 42(5), S. 814–823. DOI: 10.1016/j.indmarman.2013.01.006.

Kajan E., Stoimenov L. (2005): Toward an ontology-driven architectural framework for B2B. *Communications of the ACM*: 48(12), S. 60–66. DOI: 10.1145/1101779.1101810.

Kalvenes J., Basu A. (2006): Design of robust business-to-business electronic marketplaces with guaranteed privacy. *Management Science*: 52(11), S. 1721–1736. DOI: 10.1287/mnsc.1060.0570.

Kim M.S., Ahn J.H. (2007): Management of trust in the e-marketplace: The role of the buyer's experience in building trust. *Journal of Information Technology*: 22(2), S. 119–132. DOI: 10.1057/palgrave.jit.2000095.

Mahadevan B. (2003): Making sense of emerging market structures in B2B e-commerce. *California Management Review*: 46(1), S. 86–100. DOI: 10.2307/41166233.

Mahalingam A., Alyakoob M., Rahman M. (2020): Implications of priority access in markets with experts: Evidence from online marketplace lending. *41st International Conference on Information Systems*, S. 1–17.

Mallapragada G., Grewal R., Mehta R., Dharwadkar R. (2015): Virtual interorganizational relationships in business-to-business electronic markets: Heterogeneity in the effects of organizational interdependence on relational outcomes. *Journal of the Academy of Marketing Science*: 43(5), S. 610–628. DOI: 10.1007/s11747-014-0411-8.

Matook S. (2013): Measuring the performance of electronic marketplaces: An external goal approach study. *Decision Support Systems*: 54(2), S. 1065–1075. DOI: 10.1016/j.dss.2012.10.032.

Möhlmann M. (2021): Unjustified trust beliefs: Trust conflation on sharing economy platforms. *Research Policy*: 50(3), S. 1–47. DOI: 10.1016/j.respol.2020.104173.

Ondrus J., Gannamaneni A., Lyytinen K. (2015): The impact of openness on the market potential of multi-sided platforms: A case study of mobile payment platforms. *Journal of Information Technology*: 30(3), S. 260–275. DOI: 10.1057/jit.2015.7.

Oppong-Tawiah D., Bassellier G., Pinsonneault A. (2020): Tracing the next-generation platform firm: A typology of digital platforms as new organizing forms. *28th European Conference on Information Systems*, S. 1–10.

Ordanini A., Micelli S., Di Maria E. (2004): Failure and success of B-to-B exchange business models: A contingent analysis of their performance. *European Management Journal*: 22(3), S. 281–289. DOI: 10.1016/j.emj.2004.04.013.

Pavlou P.A., Gefen D. (2004): Building effective online marketplaces with institution-based trust. *Information Systems Research*: 15(1), S. 37–59. DOI: 10.1287/isre.1040.0015.

Petersen K.J., Ogden J.A., Carter P.L. (2007): B2B e-marketplaces: A typology by functionality. *International Journal of Physical Distribution and Logistics Management*: 37(1), S. 4–18. DOI: 10.1108/09600030710723291.

Pressey A.D., Ashton J.K. (2009): The antitrust implications of electronic business-to-business marketplaces. *Industrial Marketing Management*: 38(4), S. 468–476. DOI: 10.1016/j.indmarman.2008.02.012.

De Reuver M., Sørensen C., Basole R.C. (2018): The digital platform: A research agenda. *Journal of Information Technology*: 33(2), S. 124–135. DOI: 10.1057/s41265-016-0033-3.

Rolland K.H., Mathiassen L., Rai A. (2018): Managing digital platforms in user organizations: The interactions between digital options and digital debt. *Information Systems Research*: 29(2), S. 419–443. DOI: 10.1287/isre.2018.0788.

Saadatmand F., Lindgren R., Schultze U. (2017): Evolving shared platforms: An imbrication lens. *38th International Conference on Information Systems*, S. 1–20.

Smits M., Weigand H. (2010): Identifying market performance indicators that can be influenced by electronic intermediaries. *18th European Conference on Information Systems*, S. 1–13.

Standing C., Lin C. (2007): Organizational evaluation of the benefits, constraints, and satisfaction of business-to-business electronic commerce. *International Journal of Electronic Commerce*: 11(3), S. 107–134. DOI: 10.2753/JEC1086-4415110304.

Standing C., Love P.E.D., Stockdale R., Gengatharen D. (2006): Examining the relationship between electronic marketplace strategy and structure. *IEEE Transactions on Engineering Management*: 53(2), S. 297–311. DOI: 10.1109/TEM.2005.861801.

Standing S., Standing C., Love P.E.D. (2010): A review of research on e-marketplaces 1997–2008. *Decision Support Systems*: 49(1), S. 41–51. DOI: 10.1016/j.dss.2009.12.008.

Suh T., Houston M.B. (2010): Distinguishing supplier reputation from trust in buyer-supplier relationships. *Industrial Marketing Management*: 39(5), S. 744–751. DOI: 10.1016/j.indmarman.2010.02.013.

Sun H. (2010): Sellers' trust and continued use of online marketplaces. *Journal of the Association for Information Systems*: 11(4), S. 182–211. DOI: 10.17705/1jais.00226.

Thitimajshima W., Esichaikul V., Krairit D. (2018): A framework to identify factors affecting the performance of third-party B2B e-marketplaces: A seller's perspective. *Electronic Markets*: 28(2), S. 129–147. DOI: 10.1007/s12525-017-0256-3.

Tuma A. (1998): Configuration and coordination of virtual production networks. *International Journal of Production Economics*: 56–57(o. A.), S. 641–648. DOI: 10.1016/S0925-5273(97)00146-1.

Veisdal J. (2020): The dynamics of entry for digital platforms in two-sided markets: A multi-case study. *Electronic Markets*: 30(3), S. 539–556. DOI: 10.1007/s12525-020-00409-4/Published.

Wang Y., Potter A., Naim M., Beevor D. (2011): A case study exploring drivers and implications of collaborative electronic logistics marketplaces. *Industrial Marketing Management*: 40(4), S. 612–623. DOI: 10.1016/j.indmarman.2010.12.015.

Wang Y., Qu Z., Tan B. (2018): How do assurance mechanisms interact in online marketplaces? A signaling perspective. *IEEE Transactions on Engineering Management*: 65(2), S. 239–251. DOI: 10.1109/TEM.2017.2786275.

Wang Y., Zhang Y., Tao F., Chen T., Cheng Y., Yang S. (2019): Logistics-aware manufacturing service collaboration optimisation towards industrial internet platform. *International Journal of Production Research*: 57(12), S. 1–20. DOI: 10.1080/00207543.2018.1543967.

Wang S., Zheng S., Xu L., Li D., Meng H. (2008): A literature review of electronic marketplace research: Themes, theories and an integrative framework. *Information Systems Frontiers*: 10(5), S. 555–571. DOI: 10.1007/s10796-008-9115-2.

Williams K., Chatterjee S., Rossi M. (2008): Design of emerging digital services: A taxonomy. *European Journal of Information Systems*: 17(5), S. 505–517. DOI: 10.1057/ejis.2008.38.

Yoo B., Choudhary V., Mukhopadhyay T. (2007): Electronic B2B marketplaces with different ownership structures. *Management Science*: 53(6), S. 952–961. DOI: 10.1287/mnsc.1060.0685.
